# Estradiol/GPER affects the integrity of mammary duct-like structures *in vitro*

**DOI:** 10.1038/s41598-020-57819-9

**Published:** 2020-01-28

**Authors:** Yu Deng, Yoshio Miki, Akira Nakanishi

**Affiliations:** 1 Department of Molecular Genetics, Medical Research Institute, Tokyo Medical and Dental University (TMDU), Bunkyo-ku, Tokyo Japan; 2 Department of Genetic Diagnosis, The Cancer Institute, Japanese Foundation for Cancer Research, Koto-ku, Tokyo Japan

**Keywords:** Breast cancer, Cell death

## Abstract

High estrogen concentration leads to an inflammatory reaction in the mammary gland tissue *in vivo*; however, the detailed mechanism underlying its specific effects on the breast duct has not been fully clarified. We used 3D-cultured MCF-10A acini as a breast duct model and demonstrated various deleterious effects of 17-β estradiol (E2), including the destruction of the basement membrane surrounding the acini, abnormal adhesion between cells, and cell death *via* apoptosis and pyroptosis. Moreover, we clarified the mechanism underlying these phenomena: E2 binds to GPER in MCF-10A cells and stimulates matrix metalloproteinase 3 (MMP-3) and interleukin-1β (IL-1β) secretion *via* JNK and p38 MAPK signaling pathways. IL-1β activates the IL-1R1 signaling pathway and induces continuous MMP-3 and IL-1β secretion. Collectively, our novel findings reveal an important molecular mechanism underlying the effects of E2 on the integrity of duct-like structures *in vitro*. Thus, E2 may act as a trigger for ductal carcinoma transition *in situ*.

## Introduction

Estrogens are female hormones secreted mainly by ovaries. They serve diverse functions in the female reproductive organs (e.g., uterus and mammary gland) as well as bones, blood vessels, nerves, and brain^1–4^. Estrogens comprise three types: estrone (E1), 17-β estradiol (E2), and estriol (E3)^5^. E2 binds to estrogen receptors α and β, and the complex formed migrates to DNA of the target gene to activate its transcription^6^. In addition to these classic transcriptional mechanisms, G-protein-coupled receptor 30 (GPER) is an estrogen receptor involved in nongenomic mechanisms^7,8^. GPER expression is tissue-specific, with high expression found in the reproductive system, heart, intestine, ovary, CNS, pancreatic islets, adipose tissue, and neurons. And abnormal GPER expression is associated with depression, hypertension, diabetes, and osteoporosis^9–11^. Also, high GPER expression in breast cancer is associated with increased recurrence^12,13^. Estrogen action *via* GPER leads to enhanced fibronectin matrix assembly in breast cancer cells^14^. An important biological consequence of GPER activation is the regulation of cell growth and apoptotic cell death^15,16^. In this process, E2 binding to GPER causes G-protein complex dissociation^17,18^, and then the βγ subunit activates the tyrosine kinase Src, which leads to the activation of MAPK pathway^17,18^. GPER activation further activates the downstream signaling molecules such as MAPK and PI3K/AKT^19,20^.

In this study, 3D cultured MCF-10A acini were exposed to E2, which led to the disruption of basement membrane and cell death of some ductal cells. And we further revealed the underlying mechanism in which E2 binding to GPER resulted in cAMP-mediated activation of c-jun N-terminal kinase (JNK) and p38 MAPK signaling pathway, followed by interleukin 1β (IL-1β) and matrix metalloproteinase-3 (MMP-3) expression and secretion.

## Results

### Estradiol induces basement membrane disruption in MCF-10A acini

**Figure 1:**
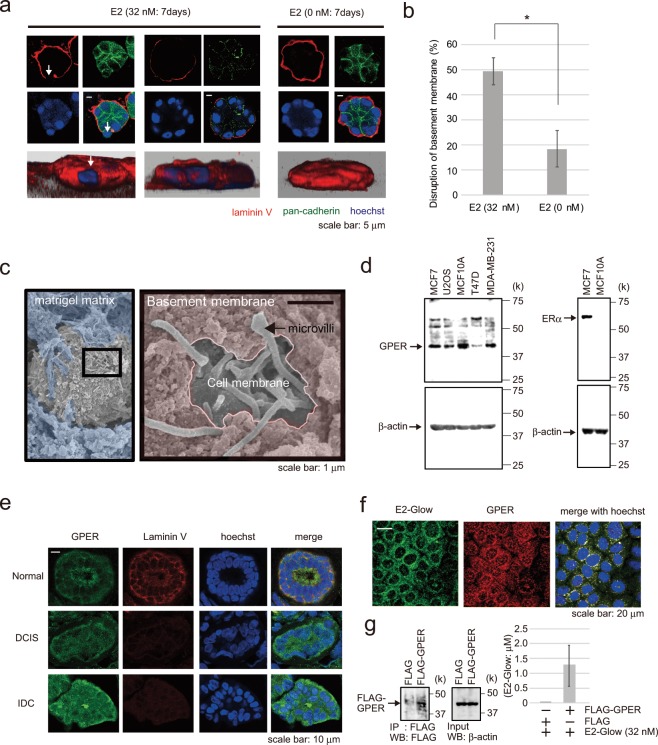
Effect of E2 on a 3D model of the milk duct using MCF-10A cells. (**a**) Representative confocal images of MCF-10A cells in a 3D culture through the middle acini, which were treated with E2 (32 nM, left two panels) or control (0 nM, right panel) for 7 days. The basement membrane was examined *via* immunofluorescence staining using laminin V antibody (red); cell junctions were evaluated using pan-cadherin antibody (green). The reconstructed images of the acini structures by confocal microscopy are shown at the bottom with Hoechst (blue) and laminin V (red) staining. Arrows indicate the collapsed portion of the basement membrane. Scale bars = 5 μm. (**b**) The basement membrane was stained using anti-laminin V antibody, and the percentage of acini with disrupted basement membranes was calculated. Three independent experiments (32 nM E2; 54.5% (n = 55), 50% (n = 48), 43.8% (n = 57), 0 nM E2; 23.1% (n = 52), 22.2% (n = 54), 10% (n = 50)) were performed. Bars represent +/−SD. DATA were analyzed using a Mann-Whitney *U* test. *p values less than 0.05 were considered statistically significant. (**c**) Representative SEM images of MCF-10A cells in a 3D culture treated with 32 nM E2 for 72 h. SEM images are shown in Matrigel matrix (blue) and basement membrane (pink). (**d**) Western blotting of GPER-expressing cell lysates (MCF-7, U2OS, MCF-10A, T47D, and MDA-MB-231) (left). MCF-7 and MCF-10A cell lysates were further probed for ERα expression. (**e)** Immunohistochemical analysis of GPER expression (green) and the basement membrane (laminin V, red) in normal human breast, ductal carcinoma *in situ*, and invasive tissue. Blue, Hoechst staining. Scale bars = 10 μm. (**f**) Immunofluorescence analysis of MCF-10A cells following treatment with fluorescently labeled E2 (green) for 5 min to examine the colocalization of E2 and GPER (red). Blue, Hoechst staining. Scale bars = 20 μm. (**g**) Binding of E2-Glow to FLAG-GPER which was expressed in 293 T cells and immunoprecipitated with FLAG antibody. 1.27 ± 0.68 μM E2-Glow was bound to FLAG-GPER. Five independent experiments were performed. Bars represent +/−SD. The presented blots were cropped. Full-length blots are presented in Supplementary Fig. 5.

We constructed a 3D model using the immortalized non-transformed mammary epithelial cell line MCF-10A to investigate the effects of E2 on the ductal structure. MCF-10A cells were cultured in 3D Matrigel, and the ductal structure was formed in ~7 days (Supplementary Fig. 1a). We verified the validity of this 3D model using four parameters: (1) formation of the cavity, (2) cell–cell adhesion, (3) cell polarity, and (4) basement membrane secretion. We observed confocal Z-stack images of the 3D model which was immunostained for centrioles, pan-cadherin, and laminin V. As a result, a cavity structure and the cell–cell adhesion molecule cadherin were confirmed in 3D model (Supplementary Fig. 1a). Cell polarity showed a certain direction, with the centrosomes located inside (Supplementary Fig. 1a), and the basement membrane immunostained with laminin V antibody surrounded the duct-like structures (Fig. 1a). In normal breast tissue, the centrosomes were located inside the breast duct and showed the same polarity as the 3D model (Supplementary Fig. 1b).

In the 3D model treated with 32 nM E2, increased partial disruption of cell-cell adhesion and basement membrane were observed compared with those in control (Fig. 1a,b). However, the disruption was not observed when treated with 32 nM 17α-estradiol, which shows no specific binding activity for GPER. And E2-Glow treatment (32 nM) disrupted the basement membrane as well as did E2 (Supplementary Fig. 2e).

In particular, p53-knockout MCF-10A cells accumulated without anoikis in the ducts, and some of the cells inside the duct were released out of the basement membrane following E2 treatment (Supplementary Fig. 1c). To better visualize the basement membrane of the glandular model, we employed SEM next. Partial basement membrane loss was observed following E2 treatment, and microvilli on the cell surface constituting the duct were detected in the gap (Fig. 1c).

Estrogens bind to the estrogen receptor and GPER^7,8^. MCF-10A cells do not express ERα but express GPER (Fig. 1d). GPER was expressed in mammary gland tissues in normal breast ducts, ductal carcinoma *in situ* (DCIS), and invasive ductal carcinoma (IDC) in immunofluorescence staining (Fig. 1e). To investigate the potential effects of estradiol on cells *via* GPER, E2-Glow—fluorescently labeled E2—was added to MCF-10A cells. Immunostaining confirmed that E2-Glow was colocalized with GPER (Fig. 1f). Furthermore, we performed E2-Glow and GPER binding experiments. E2-Glow and FLAG-GPER were reacted and immunoprecipitated with an anti-FLAG antibody. Fluorescence of the sedimentation product increased with E2-Glow concentration (Fig. 1g).

### Estradiol activates the GPER signaling pathway

**Figure 2:**
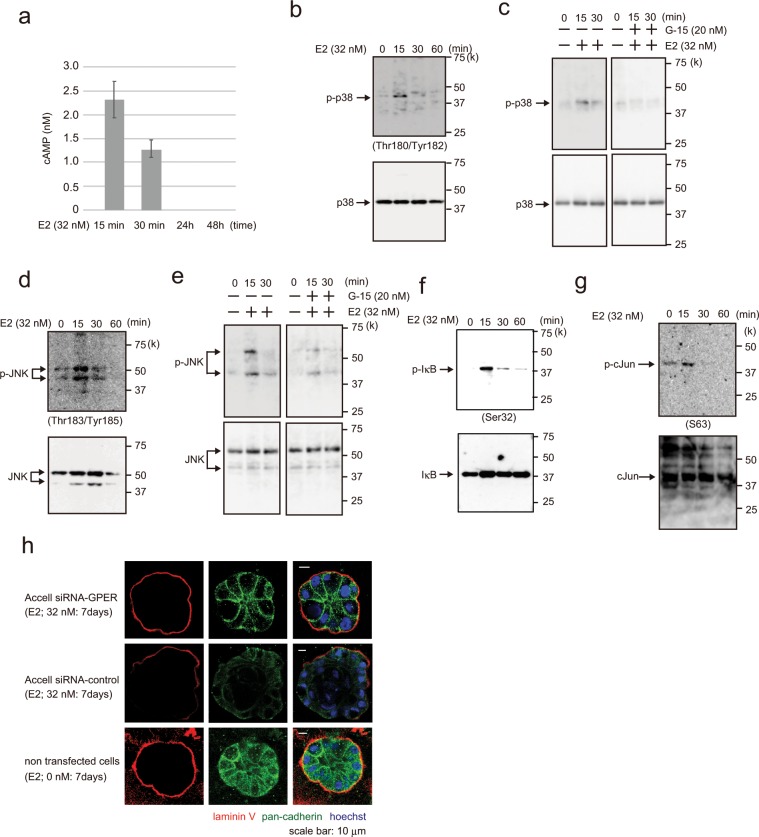
Analysis of E2 signal transduction. (**a**) cAMP assay showing cAMP levels (nM) in MCF-10A cells following treatment with 32 nM E2 for 15 min, 30 min, 24 h, and 48 h. Three independent experiments were performed. Bars represent +/−SD. (**b**) Western blotting of MCF-10A cells showing p38 and phospho-p38 (Thr180/Tyr182) following treatment with 32 nM E2 for 0–60 min. (**c**) Western blotting of MCF-10A cells treated with 32 nM E2 (left panel) or with 32 nM E2 and 20 nM G-15 (right panel) for 0–30 min. (**d**) Western blotting of MCF-10A cells showing JNK and phosphor-JNK (Thr183/Tyr185) following treatment with 32 nM E2 for 0–60 min. (**e**) Western blotting of MCF-10A cells treated with 32 nM E2 (left panel) or with 32 nM E2 and 20 nM G-15 (right panel) for 0–30 min. (**f**) Western blotting of MCF-10A cells treated with 32 nM E2 for 0–60 min showing IkB and phospho-IkB (Ser32, Ser36). (**g**) Western blotting of MCF-10A cells treated with 32 nM E2 for 0–60 min showing c-Jun and phospho-c-Jun (Ser63). (**h**) Representative confocal images of Accell siRNA-GPER- or siRNA-control-transfected MCF-10A cells in a 3D culture through the middle acini, which were treated with E2 (32 nM, left panels) or control (0 nM, right panel) for 7 days. Laminin V (red); pan-cadherin (green). Scale bars = 20 μm. The presented blots were cropped. Full-length blots are presented in Supplementary Fig. 5.

GPER activates adenylate cyclase A and induces the cAMP signaling pathway^17,21^. In this study, we verified that cAMP was activated in E2- (32 nM) and E2-Glow (32 nM)-treated MCF-10A cells (Fig. 2a, Supplementary Fig. 2a), but was not activated following 17α-estradiol (32 nM) treatment (Supplementary Fig. 2a). Furthermore, in GPER-knockdown MCF10A cells, cAMP activation was evidently reduced compared with that in control cells following E2 treatment (Supplementary Fig. 2b,c). These results suggested that E2 activated cAMP signaling *via* GPER.

Indeed, cAMP activity significantly increased at 15 and 30 min after E2 addition but showed no activity after 24 h. The p38, JNK, and IkB signaling pathways have been implicated in cAMP signaling^22,23^. To identify the signaling cascades involved in these E2-induced pathways, we examined the phosphorylation of molecules within the p38, JNK, and IkB pathways. p38, JNK, and IkB were rapidly phosphorylated following E2 stimulation, reaching a peak at 15 min and declining to the basal levels in 30 min (Fig. 2b,d,f). E2-Glow stimulation increased p38 phosphorylation, although 17α-estradiol did not induce p38 phosphorylation (Supplementary Fig. 2d). c-Jun (on Ser63), a p38 and JNK substrate, was phosphorylated to an extent similar to p38 and JNK (Fig. 2g). Moreover, we confirmed the association between GPER and the p38/JNK signaling pathway. Incubating cells with a GPER antagonist(G-15) reduced the E2-induced increases in p38 and JNK phosphorylation (Fig. 2c,e). To validate the role of GPER in E2 stimulation, we next used siRNAs to knock down GPER (Supplementary Fig. 2f–h). In control siRNA-transfected MCF-10A cells, E2 stimulation markedly increased p38 and JNK phosphorylation. However, in cells transfected with siRNA- GPER, E2-induced phosphorylation declined. We used Accell siRNA to investigate whether E2 and E2-Glow disrupted the basement membrane of 3D-cultured (>7 days) GPER-knockdown cells. And there was no basement membrane collapse observed in the Accell siRNA-GPER group as opposed to that in the siRNA-control group following E2 treatment (Fig. 2h).

Furthermore, we used MCF-7 cells expressing both ERα and GPER to examine whether E2 stimulation leads to p38 and JNK phosphorylation to a similar extent as that observed in MCF-10A cells. Phosphorylation slightly increased up to 60 min following E2 exposure (Supplementary Fig. 2i,j). These results indicated that E2-dependent p38 and JNK activation occurs *via* GPER.

### Estradiol promotes MMP-3 secretion by MCF-10A cells, and basement membrane disruption is rescued by an MMP-3 inhibitor

**Figure 3:**
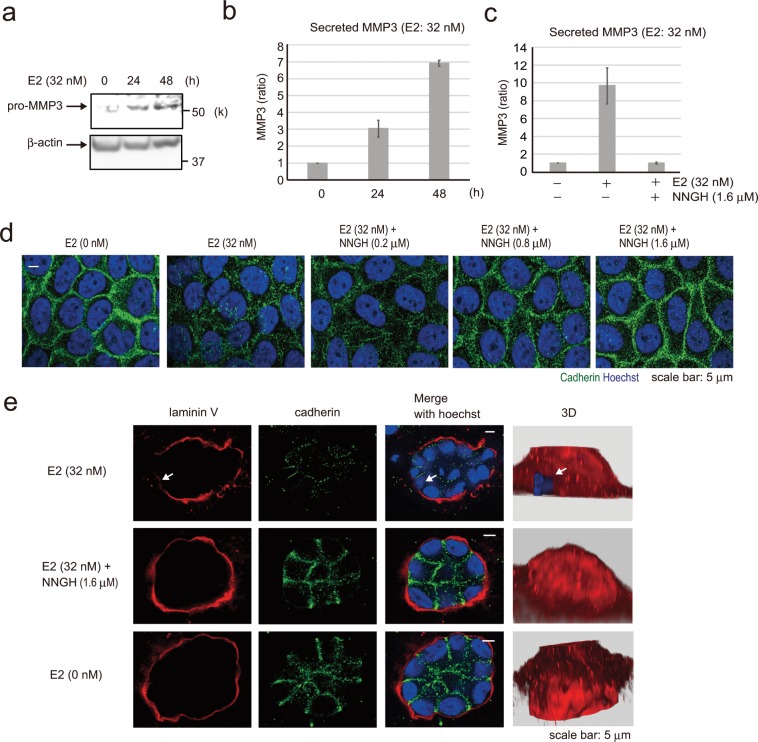
Effect of E2-induced MMP-3 secretion on cell adhesion and basement membrane. **(a**) Western blotting of MCF-10A cells showing pro- MMP-3 following treatment with 32 nM E2 for 0, 24, and 48 h. The presented blots were cropped. Full-length blots are presented in Supplementary Fig. 7. (**b**) MMP-3 activity assay of MCF-10A cells for measuring MMP-3 activity in cell culture media following treatment with 32 nM E2 for 0, 24, and 48 h. Four independent experiments were performed. Bars represent +/−SD. (**c**) MMP-3 activity assay of MCF-10A cells following treatment with 32 nM E2 with or without 1.6 μM N-isobutyl-N-(4-methoxyphenylsulfonyl)-glycylhydroxamic acid (NNGH), which is a MMP-3 inhibitor. Four independent experiments were performed. Bars represent +/−SD. (**d**) Representative images of MCF-10A cells treated with 32 nM E2 or 32 nM E2 and NNGH at 200, 800, or 1600 nM for 3 days to detect cell junctions by immunofluorescence using the pan-cadherin antibody (green). Blue staining, Hoechst. Scale bars = 5 μm. (**e**) Confocal images of MCF-10A cells in a 3D culture treated with 32 nM E2 (first row) or 32 nM E2 and 1600 nM NNGH (second row) for 14 days to investigate the basement membrane *via* staining with laminin V antibody (red) and cell junctions *via* the pan-cadherin antibody (green). Reconstruction structures of the acini are shown in the right panel by Hoechst (blue) and laminin V (red). Arrows indicate the collapsed portion of the basement membrane. Scale bars = 5 μm.

E2 stimulation disrupted cell–cell adhesion (cadherin) and the basement membrane (laminin V) in the 3D MCF-10A model (Fig. 1a). Cadherin and laminin V are the targets of MMP-3—a member of the MMP family of extracellular proteases^24,25^. Furthermore, MMP-3 is a target gene of the transcription factor AP-1, which is located downstream of JNK and p38^26–29^. Therefore, we considered that E2 may induce MMP-3 secretion. To determine the involvement of MMP-3 in the collapse of cell–cell adhesion and basement membrane following E2 stimulation, we tested the effects of E2 on pro-MMP-3 expression in cultured MCF-10A cells. Cells were treated with E2 (32 nM) for 24 and 48 h, and pro-MMP-3 expression was analyzed *via* western blotting (Fig. 3a). Pro-MMP-3 expression was confirmed 24 h after E2 addition. To measure the activity of MMP-3 secreted into the medium, cells were treated with E2 for 24 and 48 h, and the media was analyzed using the MMP-3 Activity Assay Kit. The activity of secreted MMP-3 extracellularly increased in a time-dependent manner (Fig. 3b), and was suppressed following the addition of *N*-isobutyl-*N*-(4-methoxyphenylsulfonyl)-glycyl hydroxamic acid (NNGH)—a commonly used semi-selective MMP-3 inhibitor (Fig. 3c). To evaluate whether E2-induced MMP-3 secretion was responsible for the disruption of the intercellular junctions in confluent MCF-10A cells, we used immunofluorescence analysis to determine cadherin localization (Fig. 3d). And consistent with our observations in MCF-10A cells, NNGH and E2 combination could rescue adherent junctions in a concentration-dependent manner. Furthermore, cadherin degradation in siRNA-pro-MMP-3-treated MCF-10A cells was clearly suppressed compared with that in siRNA-control cells. These results suggested that E2 induces MMP-3 secretion and acts on cadherin (Supplementary Fig. 3a). Then, we investigated the effects of NNGH on basement membrane using the 3D model (Fig. 3e). Cadherin and basement membrane disappearance due to E2 exposure was restored to normal levels (E2 0 nM) by NNGH. These results suggested that the E2-induced MMP-3 secretion led to the loss of cell–cell adhesion and basement membrane in 3D model.

### Estradiol induces MCF-10A cells pyroptosis

**Figure 4:**
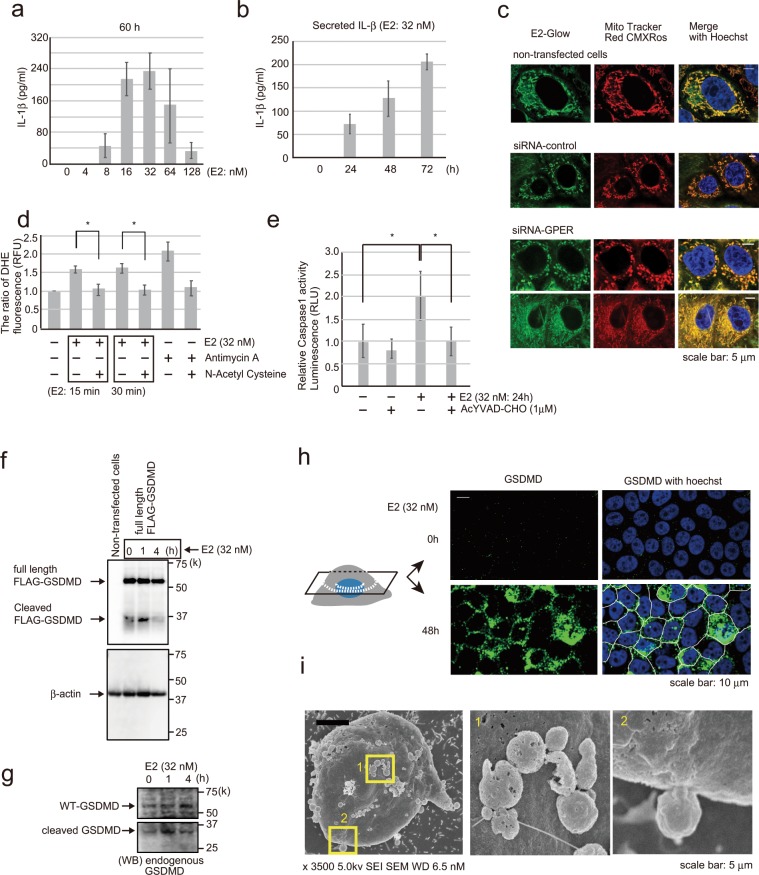
E2-induced IL-1β secretion and pyroptosis. (**a**) IL-1β ELISA of MCF-10A cells examined for the concentrations of secreted IL-1β in the cell culture media following treating the cells with 0–128 nM E2 for 60 h. Four independent experiments were performed. Bars represent +/−SD. (**b**) IL-1β ELISA of MCF-10A cells showing the concentrations of secreted IL-1β in the supernatant following treatment with 32 nM E2 for 1–72 h. Three independent experiments were performed. Bars represent +/−SD. (**c**) Representative immunofluorescence images of MCF-10A cells following treatment with fluorescently labeled E2 (green) and MitoTracker Red CMXRos (red) to examine the localization of E2 in mitochondria. E2 localized to mitochondria with or without GPER. Blue staining, Hoechst. Scale bars = 5 μm. (**d**) Cell-based ROS assay to measure ROS in MCF-10A cells following treatment with 32 nM E2 for 15 or 30 min. Antimycin A, an inhibitor of complex 3 of the mitochondrial electron transport chain, was included as a positive control for ROS production, and N-acetyl cysteine was included as an antioxidant control. Four independent experiments were performed. Bars represent +/−SD. DATA were analyzed using a Mann-Whitney *U* test. *p values less than 0.05 were considered statistically significant. (**e**) Caspase-1 inflammasome assay was used to measure caspase-1 activity in MCF-10A cells after adding 32 nM E2 for 24 h. YVAD-CHO was used as a caspase-1 inhibitor. Three independent experiments were performed. Bars represent +/−SD. DATA were analyzed using a Mann-Whitney *U* test. *p values less than 0.05 were considered statistically significant. (**f**) Western blotting of MCF-10A cells transfected with p3xFLAG-GSDMD to investigate full-length FLAG-GSDMD and cleaved FLAG-GSDMD (31 kDa) using the FLAG antibody following treatment with 32 nM E2 for 0–4 h. (**g**) Western blotting of MCF-10A cells showing endogenous WT-GSDMD (full length) and cleaved GSDMD following treatment with 32 nM E2 for 0–4 h using an antibody that recognizes the GSDMD-N-terminal. (**h**) Confocal images of MCF-10A cells treated with 32 nM E2 for 48 h (bottom) or without 32 nM E2 (up) and stained with the GSDMD antibody (green) to investigate GSDMD (N-terminal) distribution on the plasma membrane. Two confocal cellular cross sections are shown. Scale bars = 10 μm. (**i**) SEM electron microscopy imaging of MCF-10A cells treated with 32 nM E2 for 72 h showing pyroptotic bodies on the surface of the plasma membrane. Scale bars = 1 μm. The presented blots were cropped. Full-length blots are presented in Supplementary Fig. 7.

IL-1β is secreted by fibroblasts expressing GPER *via* E2^30,31^. Therefore, we next examined whether MCF-10A cells exposed to E2 secreted IL-1β into the medium (Fig. 4a). IL-1β secretion showed the highest level following 32 nM E2 addition to MCF-10A cells for 60 h. IL-1β secretion in the medium increased with time and was detected for up to 120 h (Fig. 4b, Supplementary Fig. 3b). IL-1β is expressed as pro-IL-1β by various transcription factors, including AP-1 and NFκB^32–34^. Pro-IL-1β is then processed by caspase-1 activated *via* inflammasome to become mature IL-1β^35–37^. Caspase-1 activation *via* inflammasome is induced by reactive oxygen species (ROS) production^38,39^. Therefore, we investigated whether E2 induces ROS production and caspase-1 activation. Fluorescence-labeled E2 and MitoTracker probe for labeling live cell mitochondria were added to MCF-10A cells, and the cells were observed after 20 min. E2 accumulation in the mitochondria was confirmed (Fig. 4c). Moreover, ROS production was detected at 15 min following exposure to E2 (32 nM), and was reduced after the addition of a negative control antioxidant (N-acetyl cysteine) (Fig. 4d). The experimental system functioned normally under increased ROS production after the addition of positive control (antimycin A). Mitochondrial ROS activate caspase-1 *via* the NLRP3 inflammasome^40,41^. In the current study, E2 (2 and 32 nM) activated caspase-1 (Fig. 4e, Supplementary Fig. 3c), and the activity was suppressed by the caspase-1-specific inhibitor Ac-YVAD-CHO.

Activated caspase-1 cleaves GSDMD, which then forms a complex and opens a membrane hole in the cell membrane^42–45^. IL-1β is then secreted out of the cell through those membrane pores^46^. Our results showed that E2 (32 nM) converted wild-type FLAG-GSDMD in MCF-10A cells to cleaved FLAG-GSDMS in 1 h (Fig. 4f). The cleavage product matched the size of the N-terminal fragment in cells. Then we confirmed that endogenous GSDMD was cleaved similar to FLAG-GSDMD in E2-stimulated MCF-10A cells (Fig. 4g). We next used confocal immunofluorescence microscopy to visualize the cellular distribution of endogenous GSDMD using an anti-N-terminal GSDMD recognition antibody following E2 stimulation. The cleaved N-terminal GSDMD was localized on the cell membranes which not subjected to membrane permeabilization (Fig. 4h). The presence of morphological structures, termed pyroptotic bodies, in cells undergoing E2 stimulation has been reported^47^. Pyroptotic bodies show a similar diameter (1–5 μm) to apoptotic bodies. SEM and phase-contrast microscopy revealed that E2-treated MCF-10A cells progressed to form protrusions of similar sizes as pyroptotic bodies (Fig. 4i, Supplementary Fig. 3d). These results confirmed that E2-treated MCF-10A cells secreted IL-1β and undergo pyroptosis.

### Secreted IL-1β activates the IL-1R1 signaling pathway and induces MCF-10A acini disruption

**Figure 5:**
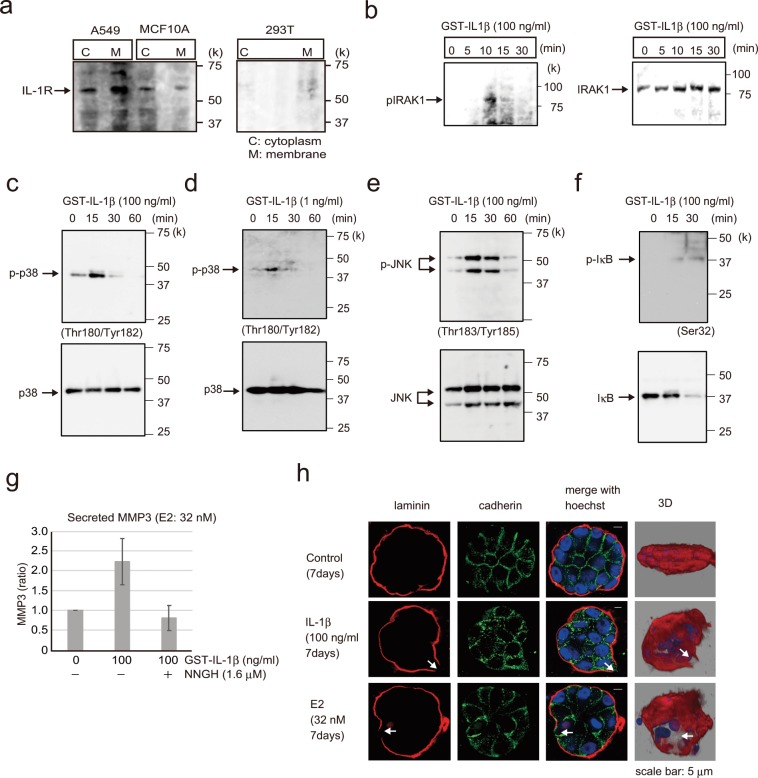
Analysis of E2 signal transduction. (**a**) Western blotting of A549, MCF-10A, and 293T cell lysates showing IL-1R expression; cytoplasmic and membrane components of the cell lysates were separated. A549 cells were included as a positive control for IL-1R1 expression, and 293T cells were used as a negative control. (**b**) Western blotting of MCF-10A cells showing phospho-IRAK1 (left) and IRAK1 (right) following treatment with 100 ng/ml GST-IL-1β for 0–30 min. (**c**) Western blotting of MCF-10A cells probed to examine phospho-p38(Thr180/Tyr182) and p38 after adding 100 ng/ml GST-IL-1β for 0–60 min. (**d**) Western blotting of MCF-10A cells probed to examine phospho-p38 (Thr180/Tyr182) and p38 after adding 1 ng/ml GST-IL-1β for 0–60 min. (**e**) Western blotting of MCF-10A cells showing phospho-JNK(Thr183/Tyr185) and JNK following treatment with 100 ng/ml GST-IL-1β for 0–60 min. (**f**) Western blotting of MCF-10A cells showing phospho-IkB (Ser32) and IkB following treatment with 100 ng/ml GST-IL-1β for 0–30 min. (**g**) MMP-3 activity assay of MCF-10A cells following treatment with 100 ng/ml GST-IL-1β with or without 1.6 μM NNGH, which was used as an MMP-3 inhibitor. Four independent experiments were performed. Bars represent +/−SD. (**h**) Representative confocal images of MCF-10A cells in a 3D culture treated with 100 ng/ml GST-IL-1β (second row) or E2 (32 nM, third row) or control (first row) for 7 days to examine the basement membrane *via* immunofluorescence staining with laminin V antibody (red) and cell junctions with the pan-cadherin antibody (green). Reconstruction images of the acini structures *via* confocal microscopy are shown in the right panel with Hoechst (blue) and laminin V (red). Arrows indicate the collapsed portion of the basement membrane. Scale bars = 5 μm. The presented blots were cropped. Full-length blots are presented in Supplementary Fig. 7.

E2 stimulation led to IL-1β secretion in MCF-10A cells (Fig. 4a,b). Next, we examined whether IL-1β secretion by E2-treated MCF-10A cells was directly related to IL-1R expression. To this end, IL-1R protein levels in MCF-10A cells were examined for the presence of fractionated cytoplasm and nuclei by western blotting (Fig. 5a). A549 cells were used as IL-1R-expressing controls and 293T cells as IL-1R-non-expressing controls. We confirmed that IL-1R was expressed in A549 and MCF-10A cells but not in 293T cells.

We next investigated the functional significance of IL-1β–IL-1R interaction. Immunofluorescence confocal microscopy revealed that IL-1β and IL-1R were colocalized in MCF-10A cells (Supplementary Fig. 4). To investigate whether IL-1β and IL-1R binding activates various downstream signals, IRAK1, p38, JNK, and IκB phosphorylation was examined (Fig. 5b–f). Phosphorylation was detected within 10 to 30 min following IL-1β (100 ng) addition. E2 (32 nM) induced IL-1β secretion (~1 ng/ml) in MCF-10A cells after 120 h (Supplementary Fig. 3b), and p38 was phosphorylated at this IL-1β concentration (Fig. 5c). To measure the activity of the MMP-3 secreted by IL-1β into the medium, cells were treated with IL-1β for 48 h, and the media were analyzed using the MMP-3 Activity Assay Kit. Activity of extracellularly secreted MMP-3 was detected, which was suppressed by the MMP-3 inhibitor NNGH (Fig. 5g). Furthermore, IL-1β (100 ng/ml) induced the disruption of basement membrane and cadherin in the 3D-cultured MCF-10A duct model, similar to E2 (Fig. 5h).

### Estradiol induces MCF-10A cells apoptosis

**Figure 6:**
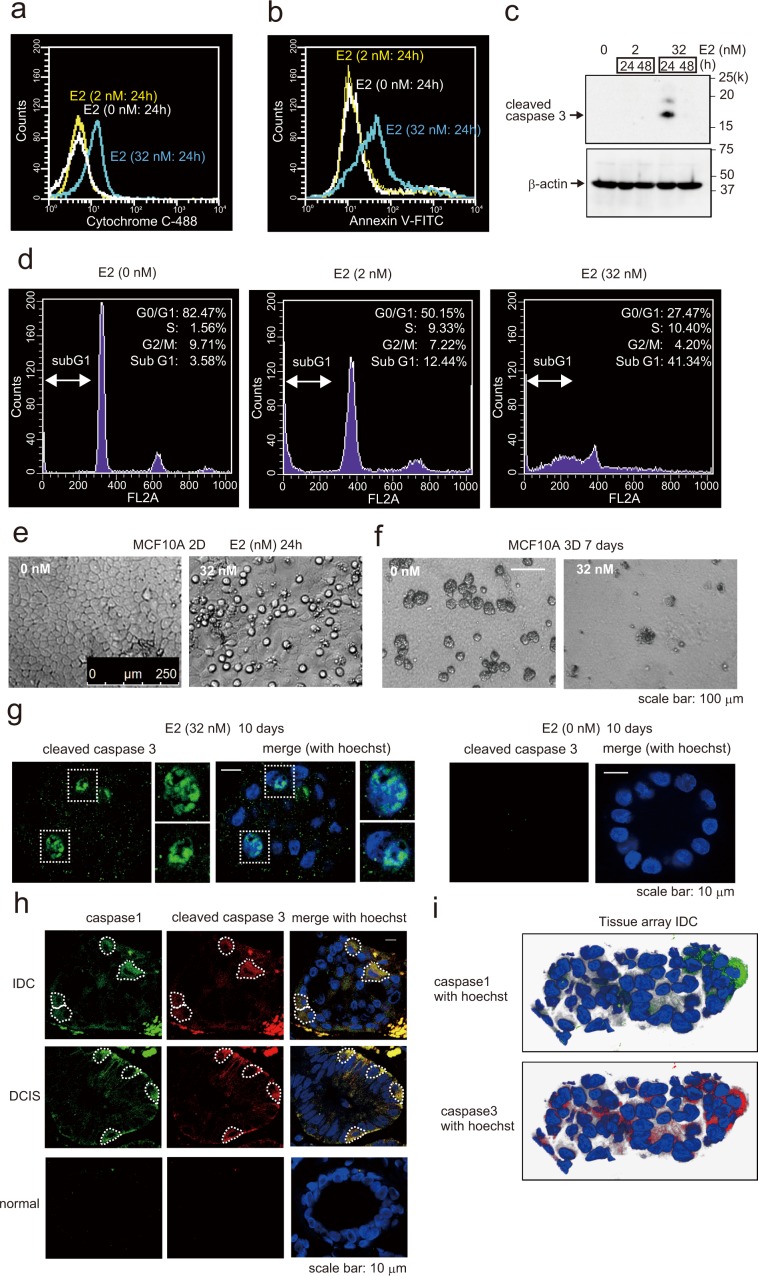
Detection of apoptosis and pyroptosis following E2 exposure. (**a**) Flow cytometry of MCF-10A cells treated with or without E2 (2 nM or 32 nM) for 24 h to examine cytochrome c expression compared with the control group. (**b**) Flow cytometry of MCF-10A cells treated with or without E2 (2 nM or 32 nM) for 24 h and stained with annexin V/PI and examined for the proportion of cells simultaneously positive for both PI and annexin V compared with the control group. (**c)** Western blotting of MCF-10A cell lysates probed to detect cleaved caspase-3 following treatment with 2 or 32 nM E2 for 24 or 48 h. (**d)** Flow cytometry of MCF-10A cells treated with or without E2 (2 or 32 nM) for 24 h to investigate cell cycle changes. (**e**) Phase-contrast morphology of MCF-10A cells grown as a monolayer and treated with or without 32 nM E2 for 24 h. (**f**) Phase-contrast micrographs of MCF-10A acini basement membranes cultured for 2 weeks and then treated with 32 nM E2 for 7 days (right). Normal MCF-10A acini (left) possess a spherical architecture similar to that observed *in vivo*. (**g**) Representative confocal images of MCF-10A acini treated with 32 nM E2 (left) or control (right). Green, cleaved caspase staining; blue, Hoechst staining. Scale bars = 10 μm. (**h**) Confocal images of human breast IDC (first row), DCIS (second row), and normal (third row) tissues immunohistochemically stained with caspase-1 antibody (green) and cleaved caspase-3 antibody (red). Caspase-1 and cleaved caspase-3 were closely merged in IDC and DCIS tissues. Blue, Hoechst staining. Scale bars = 10 μm. (**i**) Reconstruction images of IDC breast tissues *via* confocal microscopy are shown. Caspase-1 staining (green) merged with Hoechst (left) and cleaved caspase-3 staining (red) merged with Hoechst (right). The presented blots were cropped. Full-length blots are presented in Supplementary Fig. 8.

IL-1β induces cellular apoptosis by releasing cytochrome c from mitochondria^48^. In the present study of E2 induced pyroptosis, we observed that E2 was accumulated in the mitochondria, and ROS production was detected (Fig. 4c,d). E2 localizes directly to mitochondria without *via* GPER. Actually, when GPER was knocked down using siRNA, E2-Glow was localized in mitochondria (Fig. 4c). ROS production is an important factor inducing apoptosis. These results suggested that E2 induced apoptosis in addition to pyroptosis. In fact, E2 elicited several responses, including the release of cytochrome c; activation of caspase-3; and an increase in the number of annexin V-positive cells, sub-G1 cells and apoptotic cells (Fig. 6a–f). Furthermore, E2 activated caspase-3 in MF10A cells that constituted the breast duct model (Fig. 6g). And in normal breast tissues, DCIS, and IDC tissues, we examined whether pyroptosis and apoptosis occurred in the same cells which form the breast duct. To this end, tissue arrays were subjected to immunostaining with the anti-caspase-1 antibody—a marker for pyroptosis—and the anti-caspase-3 antibody—a marker for apoptosis. Caspase-1 and caspase-3 activation were observed in the same cells in both DCIS and IDC (Fig. 6h,i). These data are consistent with our hypothesis that caspase-1 and caspase-3 function as downstream components of E2-stimulated pyroptosis and apoptosis signaling pathways.

## Discussion

**Figure 7:**
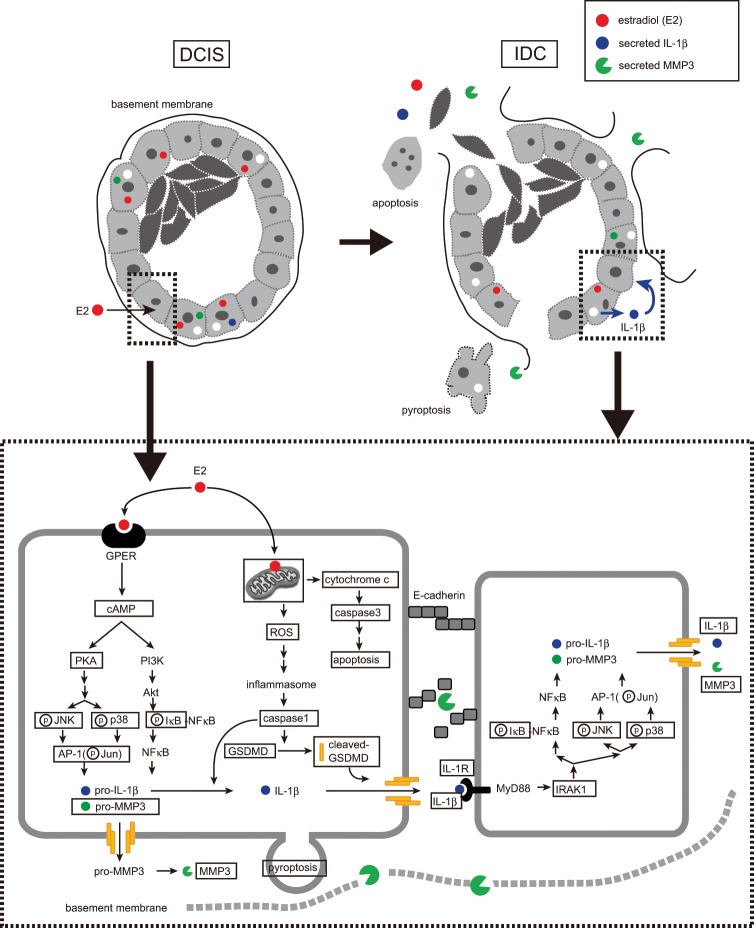
A schematic model based on our results showing the role of E2 in DCIS metastasis to IDC. E2 induced basement membrane disruption in breast glandular ducts by promoting MMP-3 and IL-1β secretion *via* the GPER signaling pathway. In turn, the secreted IL-1β activated the IL-1R1/MyD88 signaling pathway to increase IL-1β and MMP-3 expression. Finally, in our model, estradiol induces apoptosis and pyroptosis of epithelial cells, thereby disrupting the glandular ducts and promoting DCIS metastasis to IDC.

In this study, we examined the involvement of E2 in breast duct collapse. In particular, we focused on the mechanism through which E2 stimulation leads to IL-1β and MMP-3 secretion. For this purpose, we constructed a breast duct model using MCF-10A cells and evaluated the effect of E2 on this issue (Fig. 7). E2 binding to GPER led to cAMP activation and MMP-3 and IL-1β secretion. MMP-3 further degraded cadherin, which adheres to cells that make up the ducts, and laminin in the basement membrane. Moreover, E2 activated caspase-1, which degraded GSDMD to induce pyroptosis. Caspase-1 activation requires inflammasome formation, which is induced by ROS production. In this study, we also confirmed ROS production by E2. Furthermore, E2 accumulation in the mitochondria induced apoptosis along with cytochrome c release and caspase-3 activation. In contrast, IL-1β binds to IL-1R and activates various intracellular signals to promote MMP-3 secretion and induce apoptosis^49–51^. In fact, IL-1β stimulation induced MMP-3 secretion (Fig. 5g). Based on these results, E2 may trigger sequential ductal structure disruption *via* its synergistic action with IL-1β.

As a result of the genomic actions of E2 and GPER, IL-1β and MMP-3 were produced (Figs. 3b and 4a). IL-1β is elevated in various types of cancers, and IL-1β-producing tumors showed a worse prognosis in the Human Protein Atlas. IL-1β was expressed at very low levels in normal mammary epithelial cells; however, its expression was significantly elevated in DCIS and IDC^52,53^. Meanwhile, the mechanism underlying this process remains unclear. E2 concentrations in mammary gland tissues also increase with tumor progression (DCIS and IDC)^54^. Our results indicated that IL-1β secretion was significantly dependent on E2 concentration (Fig. 4a). Therefore, E2 concentration might increase with DCIS and IDC progression, thus promoting IL-1β production and eventually disrupting the ductal structure.

In this study, we showed that estrogen (32 nM) disrupted the basement membrane and ductal structure. E2 concentration in malignant breast tissue is ~1–2 pmol/g tissue^55^. If water content of the tissue is estimated to be ~60%, E2 concentration would be ~1.7–3.4 nM. As such, E2 concentration used in this study was ~10–20 times higher than the estimated E2 concentration in tissues. This poses a question of whether E2 at a concentration of 32 nM confers physiological effects. While there is no definitive answer to this question, E2 metabolites (E1, E2, and E3) are present in tissues^56,57^. For example, blood E3 concentrations of nonpregnant women are 3–19 times higher than the combined E1 and E2 concentrations^58^. Furthermore, E1 and E3 are closely involved in nongenomic GPER signaling pathways, suggesting that their role as are GPER agonists^59,60^. Therefore, comparing E2 alone in a system is not accurate. During pregnancy, blood E2 concentration increases to ≥100 nM; thus, while 32 nM E2 is considered relatively high, it is within the range of physiological conditions.

E2 binds to both GPER and ERα receptors, so how do cells presenting both receptors, such as MCF-7 cells, use each receptor properly? Because of E2 exhibits two functions (nongenomic and genomic) and E2-GPER signal transduction occurs earlier than E2-ERα signal transduction, the receptors are presumably used at different times. In MCF-10A cells (ERα^−^, GPER^+^), cAMP activation increased in 15 min following E2 addition and decreased after 30 min. After 24 h, the activity was not recognized (Fig. 2a). In addition, p38 and JNK, which are downstream of cAMP, were also phosphorylated within 30 min (Fig. 2b,d). As a nongenomic effect of E2 on MCF-7 cells, p38 and JNK phosphorylation was detected within 60 min after E2 addition (Supplementary Fig. 2i,j). In contrast, as a genomic action of E2 on MCF-7 cells, the transcriptional target gene pS2 of E2- ERα was expressed 24 h following E2 addition (data not shown). Thus, in cell types expressing both receptors, E2 appears to work synergistically *via* nongenomic and genomic actions.

In conclusion, by using a duct-like model, we clarified the mechanism through which estrogen actions destroy the ductal structure in breast cancer. Breast duct collapse may initiate breast cancer invasion, leading to cancer cell release in the breast duct as cancer progresses.

## Methods

### Cell culture

MCF-10A (non-tumorigenic epithelial cell line), MCF7, and A594 cells were purchased from ATCC. MCF-10A cells were cultured in Mammary Epithelial Cell Growth Medium (Takara) supplemented with 0.1 μg/ml cholera toxin (Sigma). MCF7 cells were cultured in MEM (Gibco) supplemented with 10% FBS (Gibco), 1% sodium pyruvate (Gibco), 1% glutamine (Gibco), and 0.1% insulin (Cell Science Technology). A594 cells were cultured in DMEM supplemented with 10% FBS. MCF-10A and TP53 (^−/−^) cells were purchased from horizon. MCF-10A and TP53 (^−/−^) cells were cultured in DMEM/F-12 medium (Gibco) containing 2.5 mM L-glutamine and 15 mM HEPES (Gibco), supplemented with 5% horse serum (Gibco), 0.5 μg/ml hydrocortisone (Sigma), 10 μg/ml human insulin (R&D System), 20 ng/ml hEGF (Sigma), and 0.1 μg/ml cholera toxin (Sigma). MDA-MB-231, T47D, U2OS, and 293T cells were generously provided by the Japanese Foundation for Cancer Research. MDA-MB-231 cells were cultured in DMEM/F-12 (Sigma). T47D cells were cultured in RPMI 1640 (Sigma) supplemented with 10% FBS and 0.2 unit/ml insulin. U2OS and 293T cells were cultured in DMEM supplemented with 10% FBS. Cells were cultured at 37 °C with 5% CO_2_. All cell lines were examined to be mycoplasma negative before experiments.

### Antibodies and reagents

The following antibodies were used in this study: pan-dadherin rabbit polyclonal antibody (ab16505, Abcam, 1:500 for WB); laminin-5 (γ2 chain) mouse mAb (MAB19562, Merck, 1:500 for IF); GPER rabbit polyclonal antibody (PA5-28647, Thermo Fisher, 1:1000 for WB and 1:100 for IF and IHC); p38 MAPK (D13E1) rabbit mAb (#8690, Cell Signaling Technology, 1:1000 for WB); phospho-p38 MAPK (Thr180/Tyr182) (D3F9) rabbit mAb (#4511, Cell Signaling Technology, 1:1000 for WB); SAPK/JNK rabbit polyclonal antibody (#9252, Cell Signaling Technology, 1:1000 for WB); phospho-SAPK/JNK (Thr183/Tyr185) rabbit mAb (#81E11, Cell Signaling Technology, 1:1000 for WB); cleaved caspase-3 (Asp175) rabbit polyclonal antibody (#9661, Cell Signaling Technology, 1:1000 for WB and 1:400 for IF); cytochrome C (7H8.2C12) mouse monoclonal antibody (ab13575, Abcam, 0.1–1 μg for 10^6^ cells for FCM); FLAG mAb (F3165, Sigma-Aldrich, 1:5000 for WB); β-actin mAb (AC-74, Sigma-Aldrich, 1:2000 for WB); p63 rabbit monoclonal (ab124762, Abcam, 1:200 for IHC); IRAK1 rabbit polyclonal antibody (PA5-19855, Thermo Fisher, 1:1000 for WB); phospho-IRAK1 (Thr100); rabbit polyclonal antibody (PA5-38631, Thermo Fisher, 1:1000 for WB); GSDMD (126-138) antibody produced in rabbit (G7422, Sigma, 1:500 for WB, 1:200 for IF); caspase-1 (p20) mouse mAb (AG-20B-0042, Adipogen, 1:500 for IHC); IκB-alpha (L35A5) mouse mAb (#4814, Cell Signaling Technology, 1:1000 for WB); phospho-IkB alpha (Ser32 and Ser36) monoclonal antibody (MA5-15224, Thermo Fisher, 1:1000 for WB); c-Jun mouse monoclonal antibody (MA5-15881, Thermo Fisher, 1:1000 for WB); phospho-c-Jun (Ser63) rabbit polyclonal antibody (PA5-17890, Thermo Fisher, 1:1000 for WB); FITC-Annexin V (cat. 556547, FITC-Annexin V Apoptosis Detection Kit I, BD Pharmingen, 5 μl/1 × 10^6^ cells for FCM); IL-1 Receptor I rabbit polyclonal antibody (ab106278, abcam,1:1000 for WB and 1:500 for IF); MMP-3 recombinant rabbit monoclonal antibody (45H6L22, Thermo Fisher, 1:200 for WB).

G-15 (cat. no. 14673), a GPER antagonist, was purchased from Cayman Chemical. N-isobutyl-N-(4-methoxyphenylsulfonyl)-glycylhydroxamic acid (NNGH, cat. no. 444225, Millipore) was used as an MMP-3 inhibitor. E2-Glow, fluorescently labeled E2, was purchased from Jena Bioscience^61^. 17α-estradiol was purchased from sigma.

### SiRNA treatment

siRNA oligonucleotides for human GPER (siRNA ID: s6054) and human MMP-3 (siRNA ID: s8854) used in this study were purchased from Ambion. The nonspecific negative control was purchased from Dharmacon (siRNA: D-001810-01-50). All SiRNA treatments were performed using the Lipofectamine RNAiMAX Reagent (Invitrogen) according to the manufacturer’s instructions.

Accell GPER siRNA (target sequence: CCCUCAUCUACAGCUUUCU), Accell non-targeting siRNA (target sequence: UGGUUUACAUGUCGACUAA), and Accell siRNA Delivery Media were purchased from Dharmacon, Acell siRNA reagents enable extended-duration silencing up to 30 days.

### Plasmids and transfection

To obtain the expression vector coding for human active-type IL-1β with an N-terminal GST tag, the gene was amplified by PCR and cloned into the pGEX 4T-2 plasmid (Amersham Biosciences, Piscataway, NJ, USA). The gene was then amplified by PCR using the following primers: 5′-TATGGATCCCCAGGAATTCTCGCACCT-3′ and 5′-GCGCTCGAGTTAGGAAGACACAAATTG-3′. PCR amplification was performed using 40 cycles at 94 °C for 10 s and 68 °C for 30 s. The IL-1β gene was inserted between the BamH1 and Xho1 sites of the pGEX 4T-2 plasmid, and the plasmid was verified by DNA sequencing and protein expression. Further confirmation was performed by western blotting and IL-1β ELISA. To obtain the expression vector coding for human GSDMD with an N-terminal FLAG tag, the gene (human liver) was amplified by PCR and cloned into the p3XFLAG-Myc-CMV plasmid vector (Sigma). The gene was amplified by PCR using the following primers: 5′-TTGCGGCCGCGAATTCAATGGGGTCGGCCTTTGAG-3′ and 5′-TCGACTGGTACCGATATCATGTGGGGCTCCTGGCTCAG-3′. PCR amplification was performed using 40 cycles at 98 °C for 10 s and 68 °C for 60 s. The Gasdermin-D gene was inserted between the EcoR I and EcoR V sites of the p3XFLAG-Myc-CMV plasmid, and the plasmid was verified by DNA sequencing. All transfections were performed using the Lipofectamine^TM^ 3000 Transfection Kit (Invitrogen), according to the manufacturer’s instructions.

### Bacterial expression of recombinant proteins

Recombinant GST-tagged IL-1β was overexpressed in *E. coli* BL21 (DE3) physS-competent cells using the following protocol. Briefly, cells were grown in LB medium supplemented with 100 mg/mL ampicillin at 37 °C until an OD_600_ of 0.6–0.8 was obtained. Protein expression was induced with 0.5 mM isopropyl β-D-thiogalactoside. Cells were further cultured at 37 °C for 2 h and harvested by centrifugation. For purification, cell pellets were resuspended in lysis buffer containing 50 mM Tris–HCl (pH 7.4), 400 mM NaCl, 0.5 mM EDTA, 5 mM MgCl_2_, 5% glycerol, and 1 mM DTT supplemented with 1 mg/ml lysozyme and 0.5 mM PMSF. Cells were lysed by sonication, and the lysates were clarified by centrifugation. The supernatants were then applied to a GST microspin column (cat. 28-9523-59, GE Healthcare). Proteins were eluted using 20 mM reduced glutathione (pH 8.0), and the eluted fractions were then dialyzed against phosphate-buffered saline (PBS).

### cAMP assays

MCF-10A cells were cultured in 96-well white, clear bottom plates at a density of 8 × 10^3^ cells/well. After 2 days of incubation, cells were treated with 32 nM E2 for 15 min, 30 min, 24 h, and 48 h. Following E2 treatment, the cyclic adenosine monophosphate (cAMP) assay was performed according the manufacturer’s instructions for cAMP-Glo Assay (V1501: Promega). This is a bioluminescent assay for monitoring changes in intracellular cAMP concentrations. In the assay, cells were lysed to release cAMP, followed by the addition of the cAMP Detection Solution containing protein kinase A (PKA) and a kinase substrate. The Kinase-Glo Reagent was added next to terminate the PKA reaction and detect the remaining ATP *via* a luciferase reaction. The plates were then read using a microplate reader (EnSpire; PerkinElmer). Luminescence was correlated to cAMP concentration using a cAMP standard curve.

### Quantitative IL-1β detection

The human IL-1β ELISA Kit (BMS224/2: Invitrogen), an enzyme-linked immunosorbent assay, was used for the quantitative detection of human IL-1β. MCF-10A cells were cultured in 96-well clear plates at a density of ~ 4 × 10^3^ cells/well. After 1–2 days of incubation, the medium was changed and replaced with a fresh medium containing 32 nM E2. This treatment lasted for 60, 72, 96, and 120 h. Following treatment, the supernatant was transferred to a microwell plate coated with a monoclonal antibody against human IL-1β, which the kit provided. The human IL-1β ELISA assay was performed according to the provided protocol.

### MMP-3 activity assay

The MMP-3 Activity Assay Kit (ab118972: Abcam) was used to measure MMP-3 activity in cell culture media. MCF-10A cells were cultured in 96-well clear plates at a density of ~ 8 × 10^3^ cells/well for 1 day. After that, the cells were treated with 2 or 32 nM E2 for 24 or 48 h. To directly measure MMP-3 activity, cells were transferred to a 96-well black plate. After reacting with the MMP-3 substrate (prepared according to the protocol), the plate was read at Ex/Em = 325/393 nm twice in 2 h.

### Caspase-1 inflammasome assay

The Caspase-Glo 1 Inflammasome Assay (G9951: Promega) was used to measure caspase-1 activity. MCF-10A cells were cultured in 96-well white plates with a clear bottom for 2 days. Cells were then treated with 32 nM E2 for 24 h. After equilibration at room temperature, the caspase-1 reagent was added to the blank reaction, negative control cells, and treated cells in the culture medium. The YVAD-CHO reagent was added to the other half of the plate. YVAD-CHO is a caspase-1 inhibitor. The plate was gently mixed and then incubated for 1 h. Luminescence was measured using a plate reader.

### ROS detection cell-based assay

The ROS Detection Cell-Based Assay Kit (cat. no. 601290: Cayman Chemical) was used to detect ROS production. MCF-10A cells were plated at a density of 8 × 10^3^ cells/well in black tissue-treated 96-well plates and cultured for 2 days. Then, 32 nM E2 was added for 15 and 30 min. Fluorescence was measured at an excitation wavelength of 480 nm and an emission wavelength of 570 nm.

### Immunofluorescence microscopy

Cells were washed in PBS; fixed in 3.4% formaldehyde in PBS for 10 min on ice; and sequentially permeabilized in 50%, 75%, and 95% ethanol on ice for 5 min each. Cells were blocked with donkey serum for 30 min at room temperature, followed by incubation with a primary antibody for 1 h at room temperature. Cells were then washed three times with PBS for 5 min each and incubated with Alexa-488- or Alexa-594-conjugated secondary antibody for 30 min at 37 °C. After washing twice with PBS, DNA was stained with 1 μg/mL bisbenzimide (Hoechst 33258). Samples were then examined under a TCS SP8 confocal microscope (Leica Microsystems).

### Immunohistochemical staining

Tissue arrays (HBreD060CS05 and BR486) were purchased from US Biomax. After deparaffinization and rehydration, antigen retrieval treatment was carried out in an autoclave at 121 °C for 20 min in antigen activation buffer (pH 9.0) (Nichirei Corp.). Tissue arrays were blocked in donkey serum for 30 min at room temperature, followed by incubation with primary antibody for 1 h at room temperature. Cells were then washed three times with PBS for 5 min each and incubated with Alexa-488 or Alexa-594 conjugated secondary antibodies for 30 min at 37 °C. After washing twice with PBS, DNA was stained with 1 μg/mL bisbenzimide (Hoechst 33258).

### Immunoblotting

MCF-10A cells were harvested and washed in PBS. After centrifugation, the cell pellet was suspended in sonication buffer (20 mM Tris–HCl (pH 8.0), 100 mM NaCl, and 1 mM EDTA) containing protease inhibitors (completely EDTA-free; Roche). For phosphate protein detection, the buffer also contained a phosphatase inhibitor cocktail (50 mM sodium fluoride, 10 mM β-glycerophosphate, 10 mM sodium pyrophosphate, and 1 mM activated sodium orthovanadate; Calbiochem). The cells were then lysed and centrifuged at 15000 × g for 30 min at 4 °C. The retrieved proteins were resolved by SDS–PAGE and transferred onto a PVDF membrane (Millipore). The membrane was then incubated with primary antibodies for 1 h after blocking in SuperBlock Blocking Buffer (Thermo Scientific) at room temperature. The membrane was then probed with secondary antibodies for 1 h at room temperature. Blots were developed using the Amersham^TM^ ECL Select^TM^ Western Blotting Detection Reagent (GE Healthcare) and exposed to ImageQuant LAS 500, according to the manufacturer’s instructions.

### Three-dimensional cultures

Matrigel (40 μl) (Sigma-Aldrich) was added to each well of an eight-well glass chamber slide (Thermo Fisher) and placed in a 37 °C incubator to allow the Matrigel to solidify for 30 min. MCF-10A cells were diluted in MCF-10A medium to achieve a density of 25 × 10^3^ cells/ml, and the cells were mixed with the medium containing 4% Matrigel at a 1:1 ratio. Next, 400 μl of this mixture was placed per well on top of the solidified Matrigel in each well of the chamber slide. The final overlay solution comprised 5 × 10^3^ cells/well in a medium containing 2% Matrigel. The cells were allowed to grow in a 5% CO_2_ humidified incubator at 37 °C and were re-fed with MCF-10A medium containing 2% Matrigel every 4 days.

### Flow cytometry

A flow cytometer (FACSCalibur, BD Bioscience) was used for DNA cell analysis, cytochrome c expression, and annexin V analysis. The cell preparation for DNA cell analysis was performed as follows. Briefly, MCF-10A cells were washed with PBS and fixed in 70% ethanol. The fixed cells were then washed twice with PBS and treated with RNase A at 37 °C for 30 min. Finally, the cells were stained with propidium iodide and incubated in the dark for 30 min. Samples were analyzed by flow cytometry, and 10^4^ cells were counted for each sample.

### E2-glow binding assays

FLAG-GPER or FLAG was overexpressed in 293T cells and immunoprecipitated with Dynabeads-FLAG antibody. FLAG-GPER or FLAG was bound to Dynabeads via a FLAG antibody. 10 uM E2-glow was reacted with the immunoprecipitated product at room temperature for 15 minutes. The plate was then washed three times with PBS, and the fluorescence value was measured using a microplate reader (EnSpire; PerkinElmer). A calibration curve was prepared in advance from the concentration of E2-Glow and the fluorescence value. The fluorescence value of E2-Glow reacted with FLAG was subtracted from the fluorescence value of E2-Glow reacted with FLAG-GPER, and the concentration of E2-Glow reacted with FLAG-GPER was calculated from the calibration curve.

### Statistical analysis

The data in graphs are presented as mean ± SEM of three or more independent experiments and n values described in each figure legend represent each independent trial. Data for all experiments were analyzed using a Mann-Whitney *U* test. Values of *P* < 0.05 were considered statistically significant and the degree of significance is indicated in each figurelegend. *P < 0.05.

## Supplementary information

Supplementary information.
