# Supplementary material for: Estradiol/GPER affects the integrity of mammary duct-like structures in vitro
Source: Sci Rep. 2020 Jan 28;10:1386. doi: 10.1038/s41598-020-57819-9 (PMC6987193; doi:10.1038/s41598-020-57819-9)
Supplement: Supplementary file 1 — Supplementary information. [file 41598_2020_57819_MOESM1_ESM.pdf]

Supplementary information

Estradiol/GPER affects the integrity of mammary duct-like structures *in vitro*

Yu Deng [1], Yoshio Miki[1,2]*, and Akira Nakanishi[1]

[1]Department of Molecular Genetics; Medical Research Institute; Tokyo Medical and Dental University (TMDU); Bunkyo-ku, Tokyo, Japan

[2]Department of Genetic Diagnosis; The Cancer Institute; Japanese Foundation for Cancer Research; Koto-ku, Tokyo, Japan

*Correspondence to: Yoshio Miki; Email: miki.mgen@mri.tmd.ac.jp; Tel: 81-3-5803-5825

Supplementary figure legends

Supplementary Fig. 1 A 3D model of the milk duct using MCF-10A cells. **a** Representative confocal images of MCF-10A acini. MCF-10A cells were cultured on Matrigel for 2 weeks and immunostained with an antibody against pan-cadherin (green) to detect cell junctions as well as an antibody against the centriole (red) to illustrate its apical orientation to the hollow lumen of the acini. Hoechst-stained (blue) cells represent the reconstructed image of the acini structure (first row); a continuous confocal section (1–3) through the acinus is shown in the middle. **b** Representative images of normal breast tissue stained with pan-cadherin (green), centriole (red), and Hoechst (blue). Scale bars = 10 μm. **c** Representative confocal images of MCF-10A cell ($p53^{-/-}$) acini treated with (up) or without (bottom) 32 nM estradiol for 14 days and immunostained with laminin V (red) to delineate basement membrane secretion and pan-cadherin (green) to show the cell junctions. Blue, Hoechst staining. Scale bars = 10 μm.

Supplementary Fig. 2 Inhibition of E2-dependent signaling by siRNA-GPER. **a** cAMP assay of cAMP levels (nM) in MCF-10A cells following treatment with 32 nM E2-Glow or 17a-estradiol for 30 min. Five independent experiments were performed. Bars

represent +/-SD. **b** Western blot analysis of MCF-10A cells showing GPER expression after transfection with Accell siRNA-GPER. **c** cAMP assay of cAMP levels (nM) in siRNA-GPER transfected-MCF-10A cells or siRNA-control transfected cells following treatment with 32 nM E2 for 30 min. Five independent experiments were performed. Bars represent +/-SD. DATA were analyzed using a Mann-Whitney *U* test. *p values less than 0.05 were considered statistically significant. **d** Western blot analysis of MCF-10A cells showing phospho-p38 (Thr180/Tyr182) and p38 after treated with 17a-estradiol (32nM) and E2-Glow (32nM). **e** Representative confocal images of MCF-10A cells in 3D culture through the middle acini, which were treated with 17α-estradiol (32 nM,) or E2-Glow (32 nM) for 7 days. Laminin V (red); pan-cadherin (green). Scale bars = 10 μm. **f** Western blot analysis of MCF-10A cells showing GPER expression after transfection with siRNA-GPER. **g** Western blotting of MCF-10A cells showing phospho-p38 (Thr180/Tyr182) and p38 following treatment with 32 nM E2 and transfection with siRNA-control (left panel) or with 32 nM E2 and transfection with siRNA-GPER (right panel). **h** Western blotting of MCF-10A cells showing phospho-JNK (Thr183/Tyr185) and JNK after treatment with 32 nM E2 and transfection with siRNA-control (left panel) or with 32 nM E2 and transfection with siRNA-GPER (right panel). **i** Western blotting of MCF-7 cells treated with 32 nM E2 for 0–60 min showing phospho-p38 (Thr180/Tyr182) and p38. **j** Western

blotting of MCF-7 cells showing JNK and phospho-JNK (Thr183/Tyr185) following treatment with 32 nM E2 for 0–60 min. The presented blots were cropped. Full-length blots are presented in Supplementary Fig. 6.

Supplementary Fig. 3 Induction of the time-dependent IL-1β expression and pyroptosis following E2 exposure. **a** Western blotting of siRNA-MMP-3-transfected MCF-10A cells showing pro-MMP-3 expression after treating with E2 (32 nM) for 24 h. **e** Representative images of si-RNA control-transfected or siRNA GPER-transfected MCF-10A cells treated with E2 (32 nM).Green staining, pan-cadherin; blue staining, Hoechst. Scale bars = 20 μm. **b** IL-1β ELISA of MCF-10A cells examined for the concentration of secreted IL-1β in the cell culture media after treating the cells with 32 nM E2 for 0–120 h. Four independent experiments were performed. Bars represent +/-SD. **c** The caspase-1 inflammasome assay was used to measure caspase-1 activity in MCF-10A cells after adding 2 nM E2 for 24 h. YVAD-CHO was used as a caspase-1 inhibitor. Three independent experiments were performed. Bars represent +/-SD. DATA were analyzed using a Mann-Whitney *U* test. *p values less than 0.05 were considered statistically significant. **d** Pyroptotic bodies were detected by phase-contrast microscopy 26 h after the addition of E2. The pyroptotic bodies are shown in the square. The presented blots

were cropped. Full-length blots are presented in Supplementary Fig. 7.

Supplementary Fig. 4 Detection of the interaction between GST-IL-1β and IL-1R by immunostaining. Representative confocal images of MCF-10A cells showing the colocalization of added GST-IL-1β and IL-1R by immunofluorescence staining using GST (green) and IL-1R (red) antibodies. The white line delineates the secretion of the cell nucleus. Yellow shows the colocalization of GST-IL-1β and IL-1R. Fluorescence intensity profiles along the lines were drawn in the staining patterns. GST-IL-1β and IL-1R were closely merged. Scale bars = 10 μm.

Supplementary Fig. 5

Original full length blot of figures 1d, 1g, and 2b-2g. Boxes indicated areas shown in the figures.

Supplementary Fig. 6

Original full length blot of figures supplementary 2b, 2d, and 2f-2j. Boxes indicated areas shown in the figures.

Supplementary Fig. 7

Original full length blot of figures 3a, 4f, 4g, 5b-5f, and supplementary 3a. Boxes indicated areas shown in the figures.

Supplementary Fig. 8

Original full length blot of figure 6c. Boxes indicated areas shown in the figure.

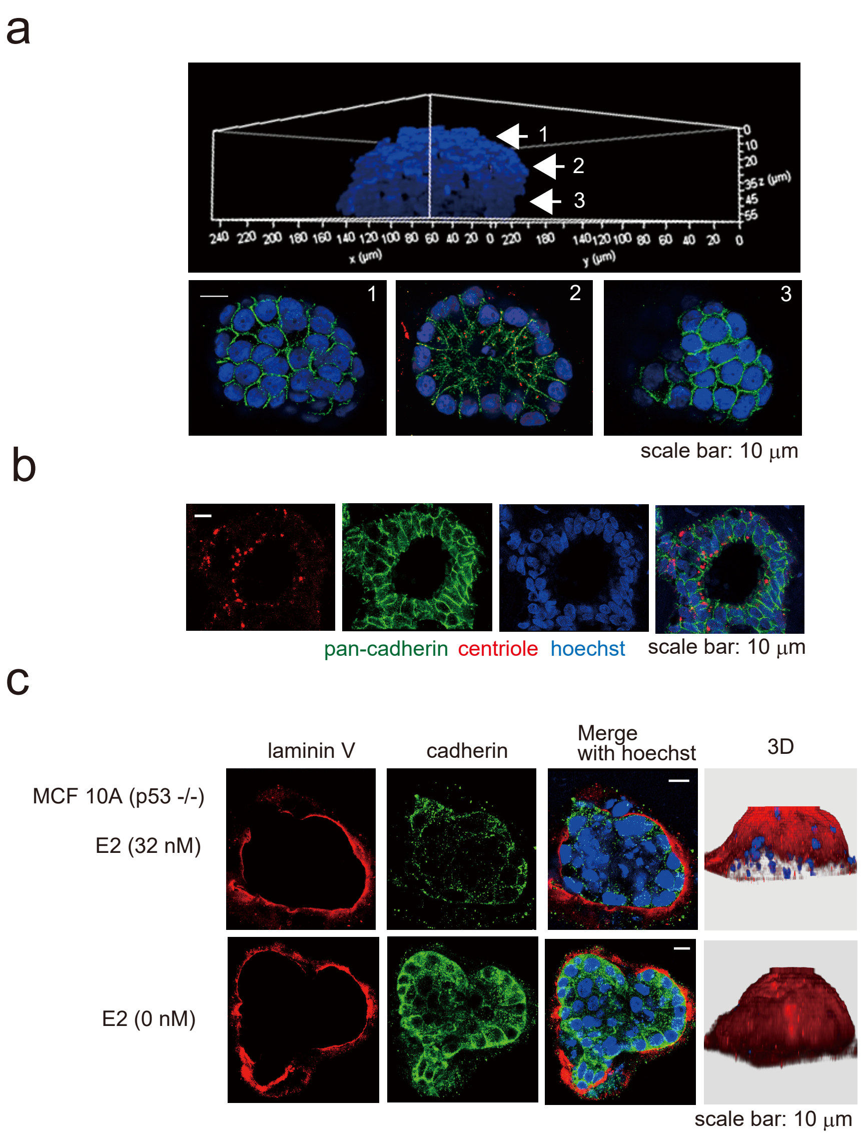
a
1
2
3
x (μm)
y (μm)
z (μm)
scale bar: 10 μm
b
pan-cadherin centriole hoechst
scale bar: 10 μm
c
laminin V
cadherin
Merge with hoechst
3D
MCF 10A (p53 -/-)
E2 (32 nM)
E2 (0 nM)
scale bar: 10 μm

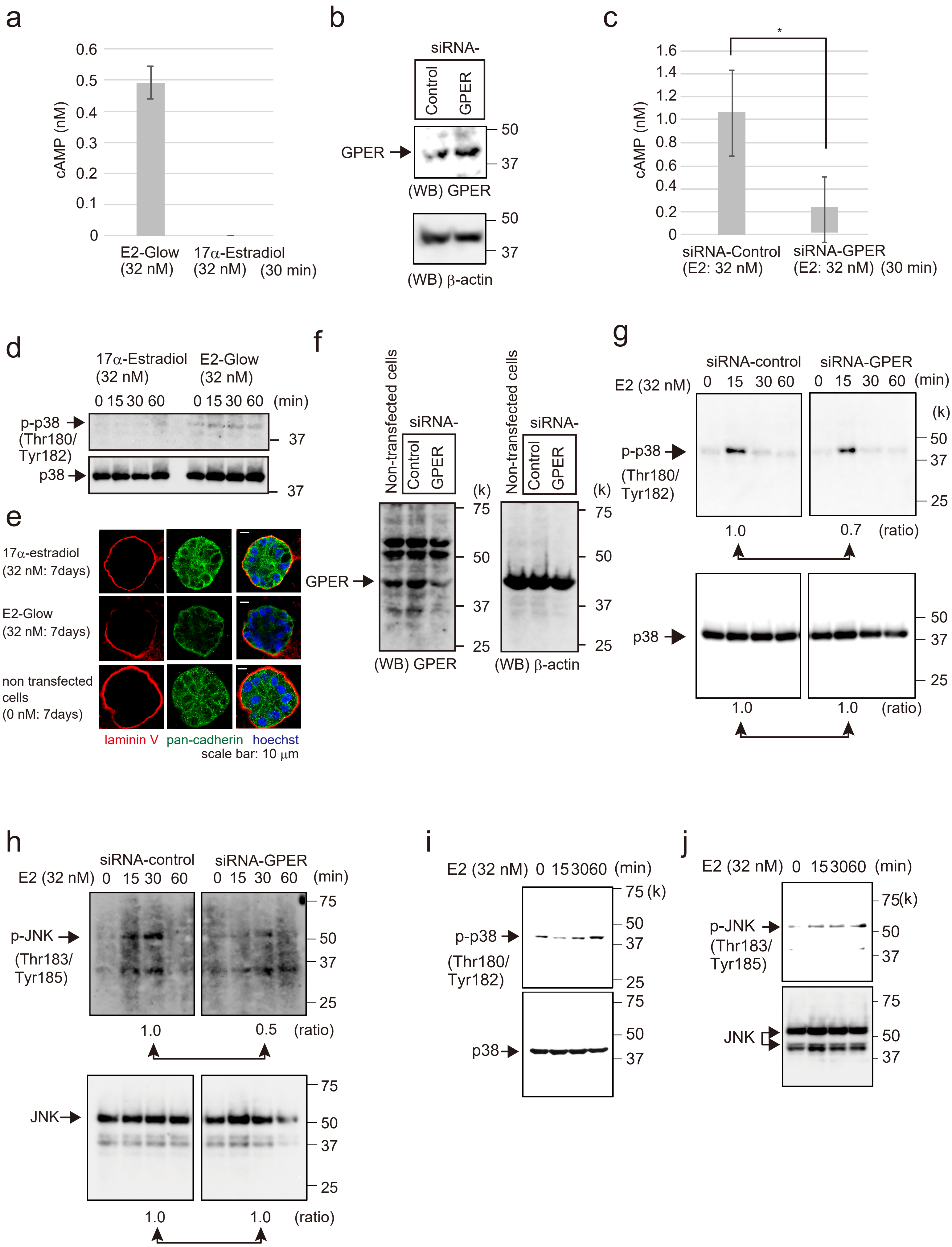
a
cAMP (nM)
0.6
0.5
0.4
0.3
0.2
0.1
0
E2-Glow (32 nM)
17α-Estradiol (32 nM)
(30 min)
b
siRNA-
Control
GPER
GPER
50
37
(WB) GPER
50
37
(WB) β-actin
c
*
cAMP (nM)
1.6
1.4
1.2
1.0
0.8
0.6
0.4
0.2
0
siRNA-Control (E2: 32 nM)
siRNA-GPER (E2: 32 nM)
(30 min)
d
17α-Estradiol (32 nM)
E2-Glow (32 nM)
0 15 30 60
0 15 30 60
(min)
p-p38 (Thr180/ Tyr182)
p38
37
37
e
17α-estradiol (32 nM: 7days)
E2-Glow (32 nM: 7days)
non transfected cells (0 nM: 7days)
laminin V
pan-cadherin
hoechst
scale bar: 10 μm
f
Non-transfected cells
siRNA-
Control
GPER
(k)
75
50
37
25
GPER
(WB) GPER
(WB) β-actin
g
siRNA-control
siRNA-GPER
E2 (32 nM)
0 15 30 60
0 15 30 60
(min)
(k)
p-p38 (Thr180/ Tyr182)
50
37
25
1.0
0.7
(ratio)
p38
1.0
1.0
(ratio)
h
siRNA-control
siRNA-GPER
E2 (32 nM)
0 15 30 60
0 15 30 60
(min)
p-JNK (Thr183/ Tyr185)
75
50
37
25
1.0
0.5
(ratio)
JNK
1.0
1.0
(ratio)
i
E2 (32 nM)
0 15 30 60
(min)
75 (k)
50
37
25
p-p38 (Thr180/ Tyr182)
p38
j
E2 (32 nM)
0 15 30 60
(min)
75 (k)
p-JNK (Thr183/ Tyr185)
50
37
JNK

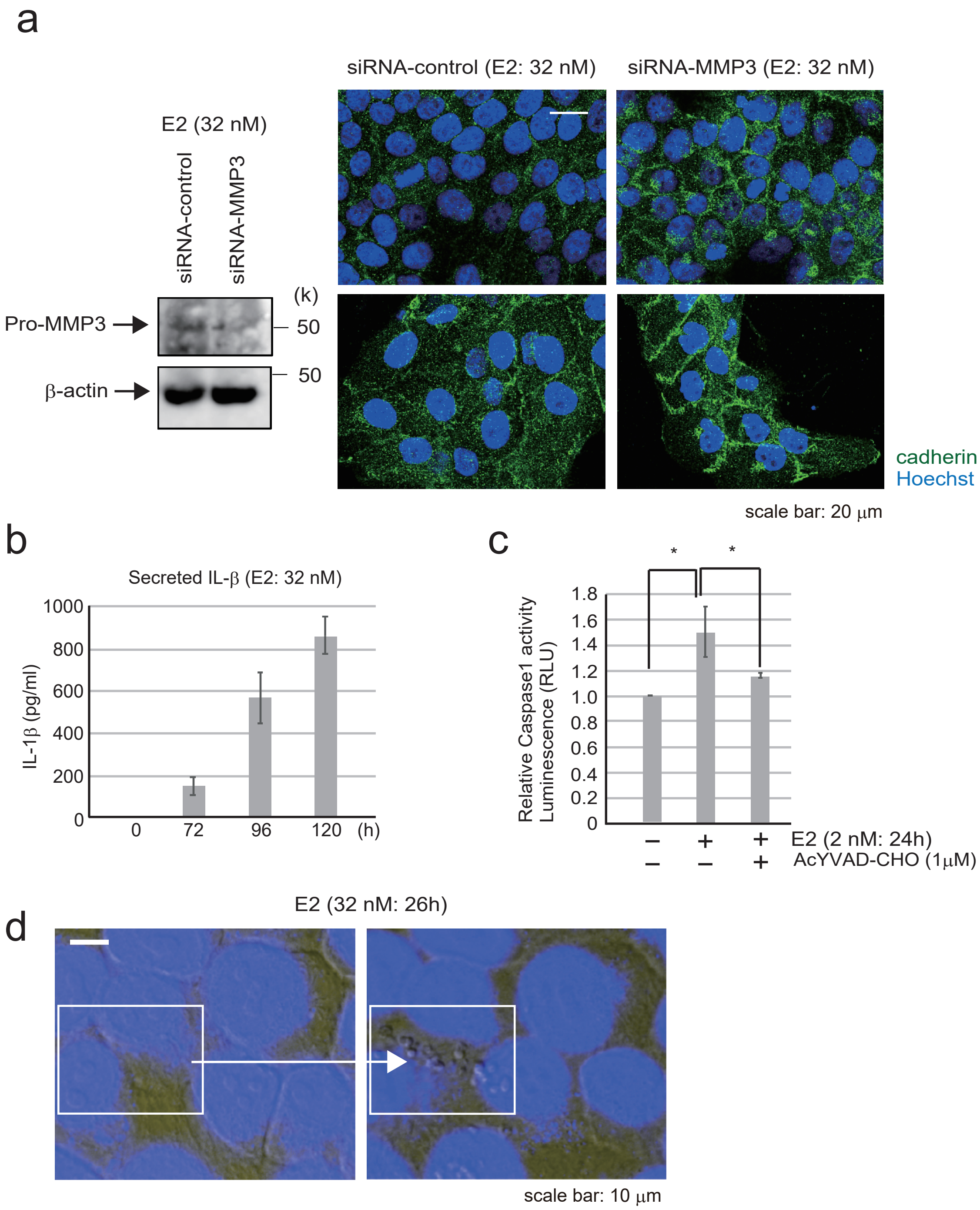
a
siRNA-control (E2: 32 nM)
siRNA-MMP3 (E2: 32 nM)
E2 (32 nM)
siRNA-control
siRNA-MMP3
(k)
Pro-MMP3
50
β-actin
50
cadherin
Hoechst
scale bar: 20 μm
b
Secreted IL-β (E2: 32 nM)
IL-1β (pg/ml)
1000
800
600
400
200
0
0
72
96
120
(h)
c
Relative Caspase1 activity Luminescence (RLU)
1.8
1.6
1.4
1.2
1.0
0.8
0.6
0.4
0.2
0
*
*
E2 (2 nM: 24h)
AcYVAD-CHO (1μM)
d
E2 (32 nM: 26h)
scale bar: 10 μm

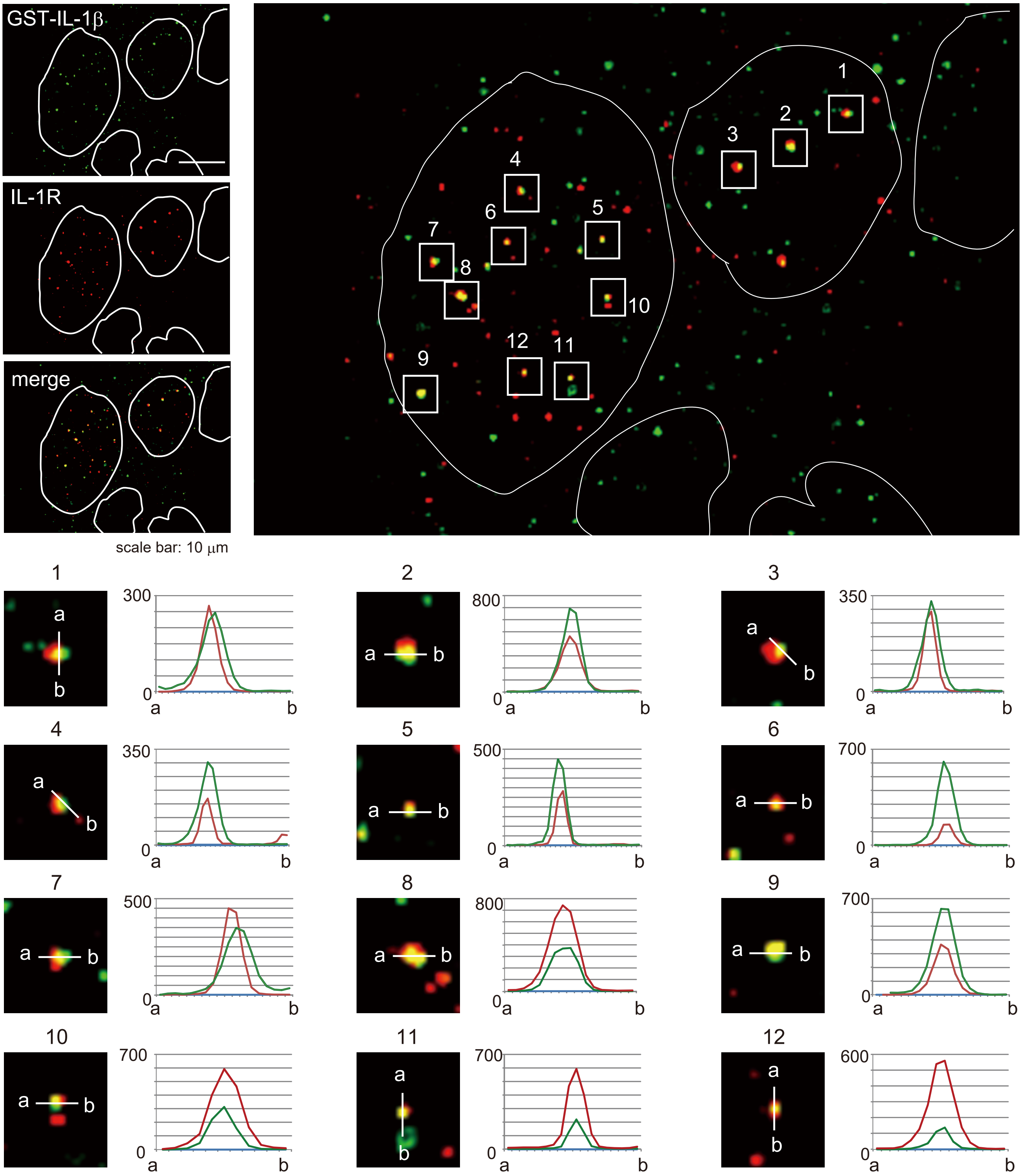

GST-IL-1β
IL-1R
merge
scale bar: 10 μm
1
2
3
4
5
6
7
8
9
10
11
12
a
b
300
350
500
600
700
800
0

Fig. 1d

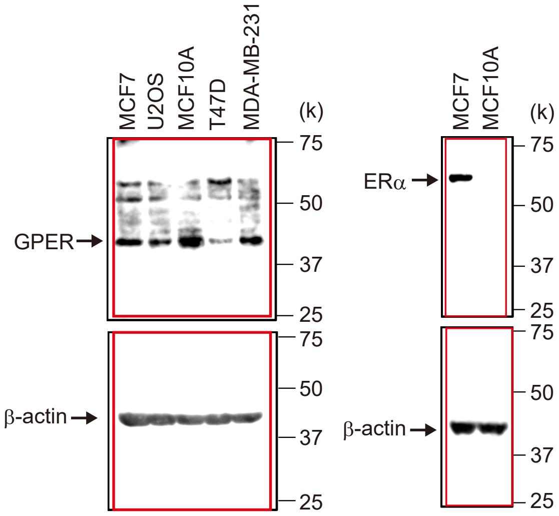

Fig. 1g

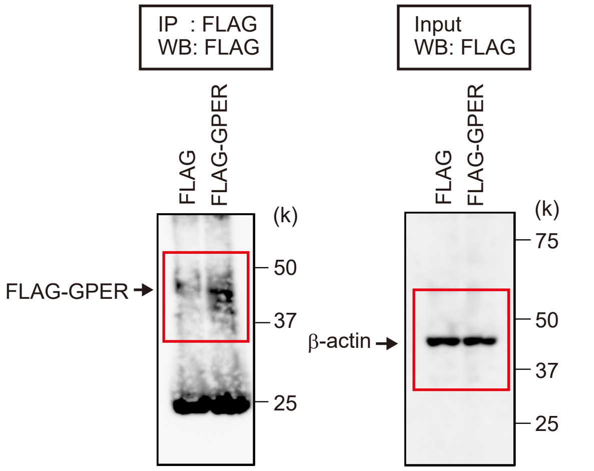

Fig. 2b

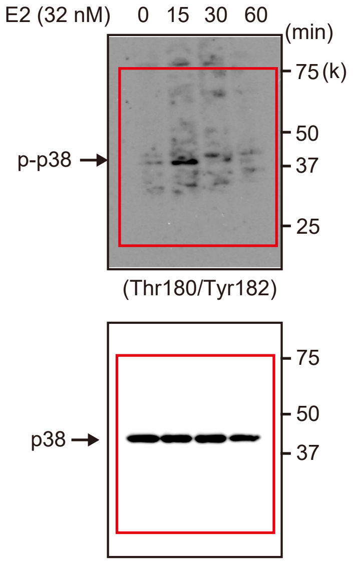

Fig. 2c

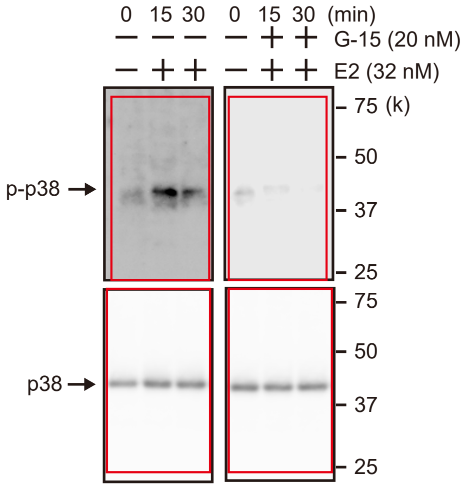

Fig. 2d

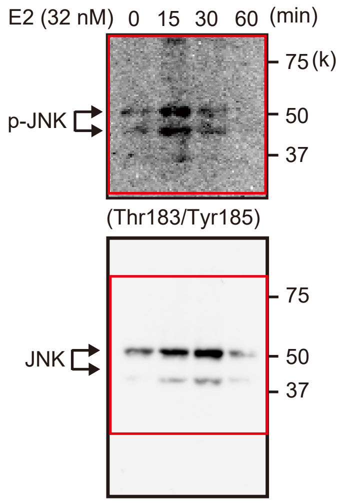

Fig. 2e

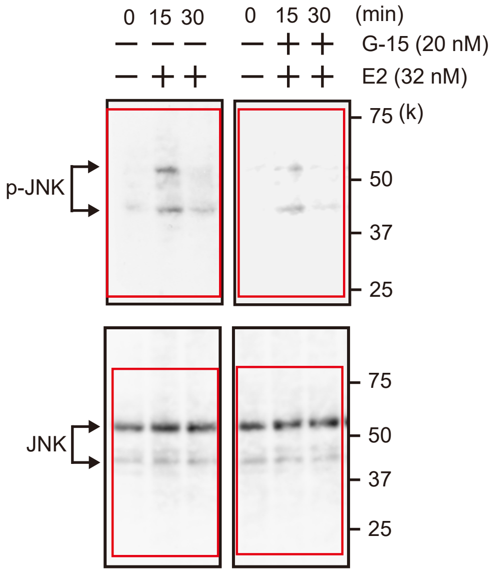

Fig. 2f

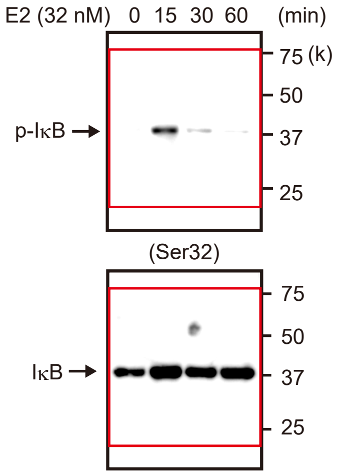

Fig. 2g

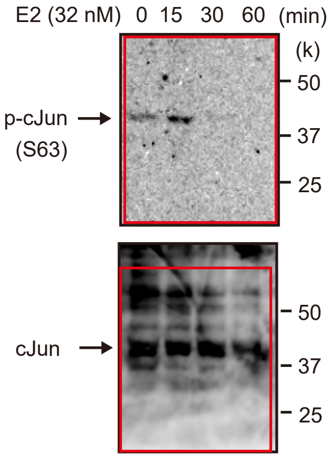

Supplemetary Fig. 2b

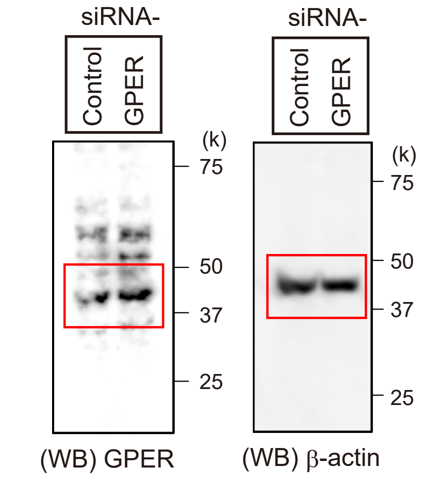

Supplemetary Fig. 2d

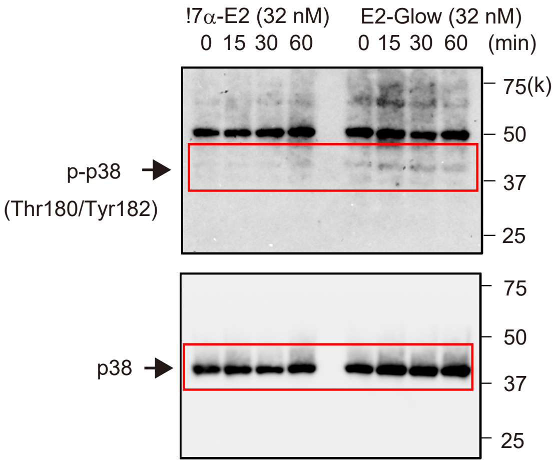

Supplemetary Fig. 2f

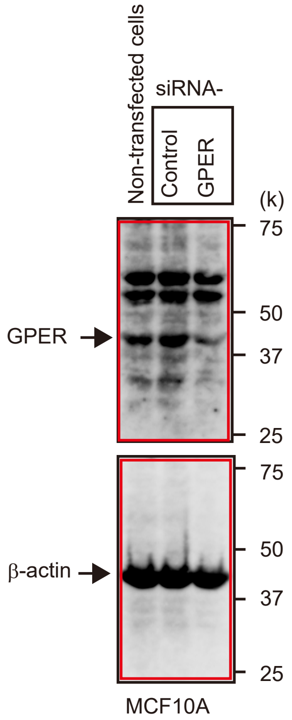

Supplemetary Fig. 2g

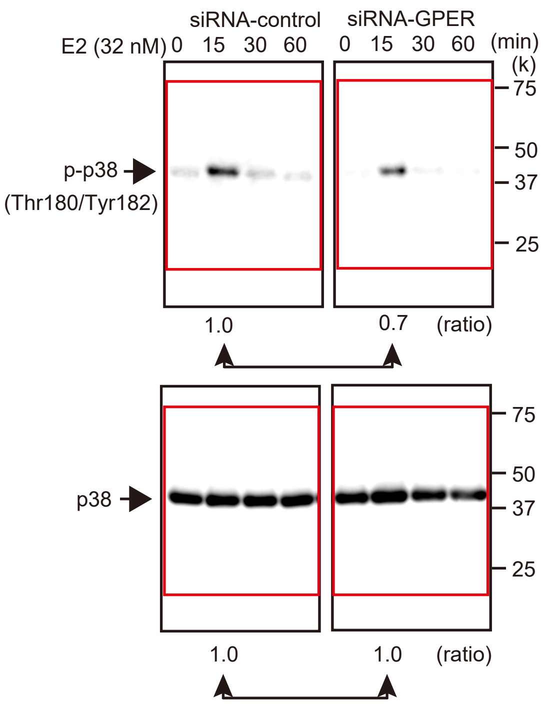

Supplemetary Fig. 2h

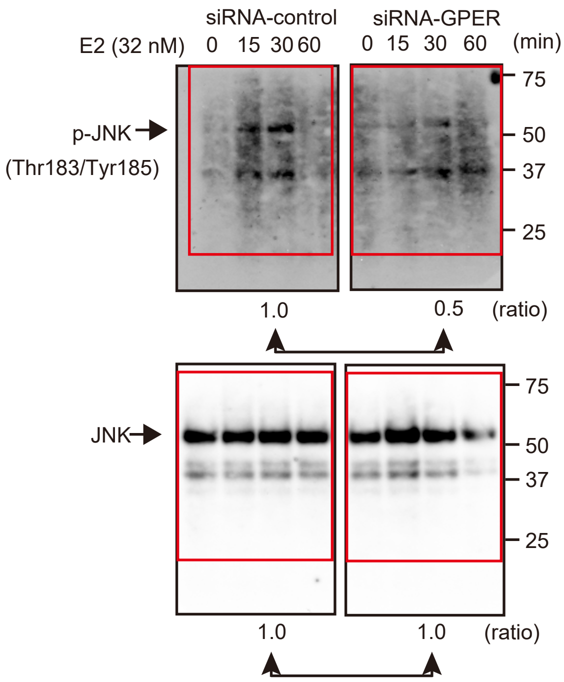

Supplemetary Fig. 2i

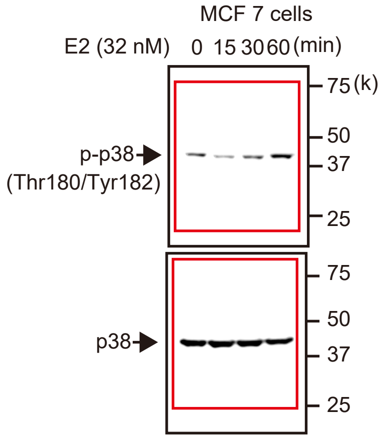

Supplemetary Fig. 2j

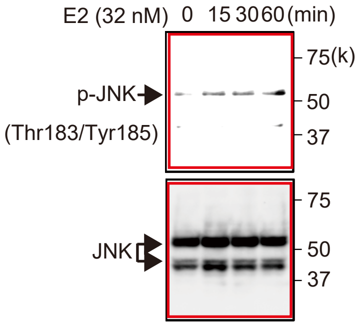

Fig. 3a

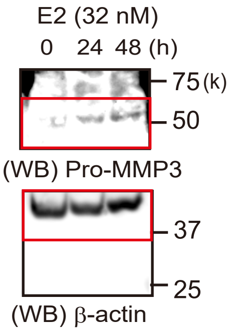

Supplemetary Fig. 3a

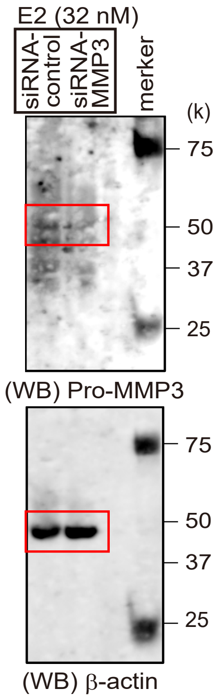

Fig. 4f

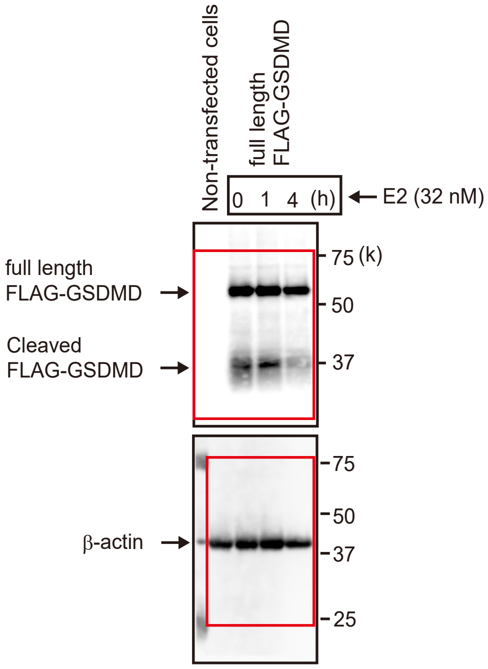

Fig. 4g

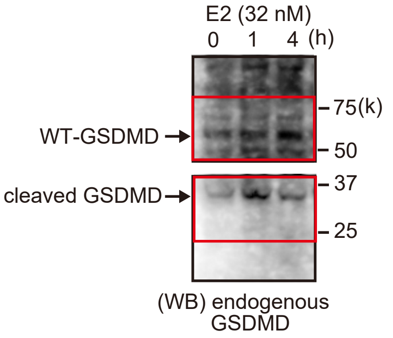

Fig. 5a

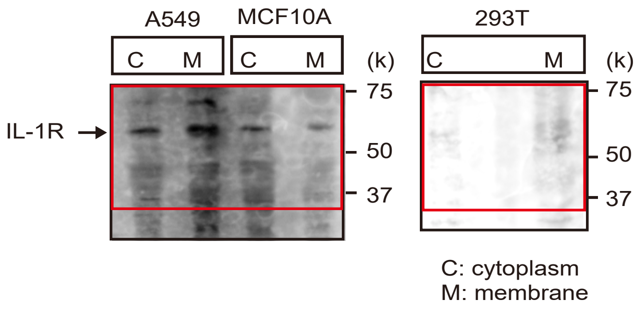

Fig. 5b

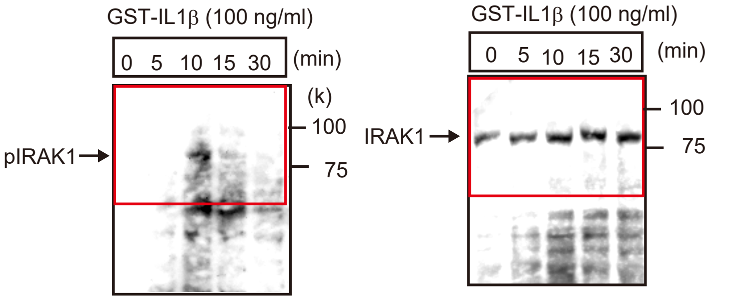

Fig. 5c

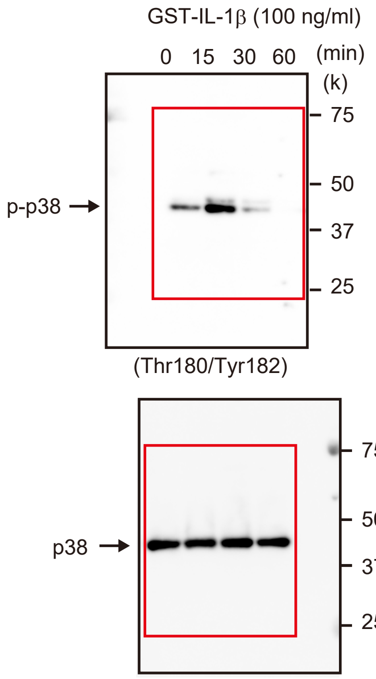

Fig. 5d

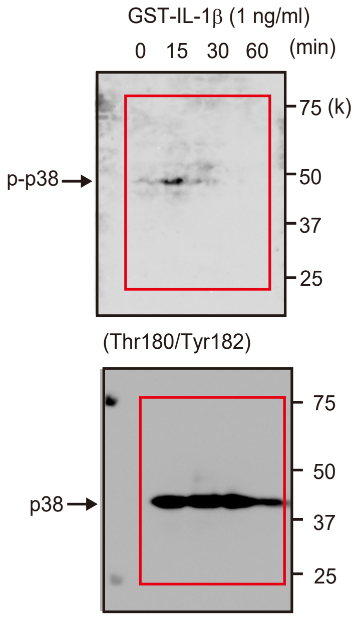

Fig. 5e

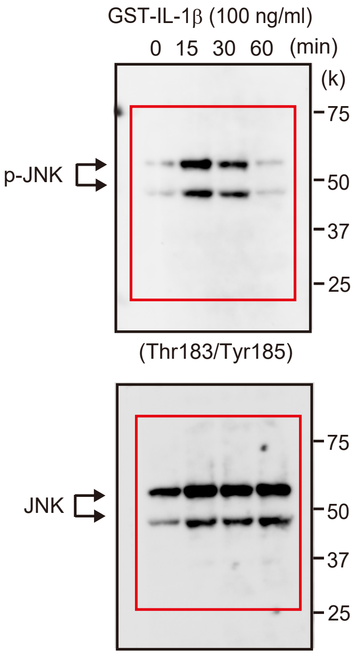

Fig. 5f

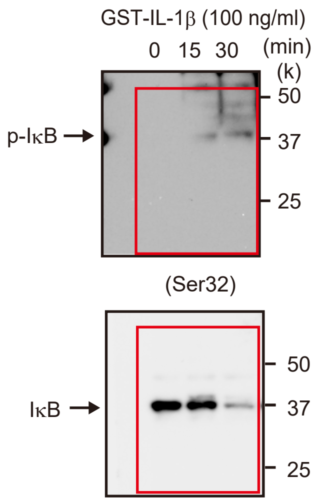

Fig. 6c

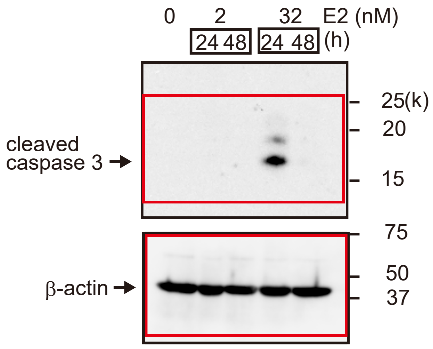
0
2
32
E2 (nM)
24 48
24 48
(h)
cleaved caspase 3
25(k)
20
15
β-actin
75
50
37
